# Chemical Synthesis of Mirror-Image Proteins Reveals Chirality-Dependent Cellular Uptake Mediated by a Cell-Penetrating Peptide

**DOI:** 10.1021/jacs.6c03792

**Published:** 2026-06-17

**Authors:** Eman Nassar, Ashok Donthoju, Mahdi Hasan, Ashraf Brik

**Affiliations:** Schulich Faculty of Chemistry Technion-Israel Institute of Technology, 3200008 Haifa, Israel

## Abstract

Mirror-image peptides and proteins are attracting interest as therapeutics, as key building blocks for constructing mirror-image life, and as tools to probe the origin of life. Their resistance to proteolytic degradation and unique stereochemistry make D-peptides/proteins particularly appealing for biomedical applications, yet a critical unresolved question is how their intracellular uptake compares to that of natural L-forms. To address this, we systematically investigated the role of cargo chirality in cellular internalization while maintaining a constant delivery vehicle. Three model cargos of increasing size and structural complexity were synthesized in both L- and D-configurations and conjugated to an identical cyclic deca-arginine (cR10) cell-penetrating peptide (CPP). By keeping the CPP scaffold constant, we reduced delivery-related variability and directly assessed the influence of the cargo chirality on uptake. Quantitative uptake analysis using flow cytometry, gel analysis, and confocal microscopy across multiple mammalian cell lines reveals that L-cargos are internalized more efficiently than their mirror image D-counterparts, demonstrating that cargo chirality is a key determinant of uptake efficiency across the chiral biological membrane. Collectively, these findings provide a systematic basis for further exploration of chirality effects in CPP-mediated delivery and may inform the design of mirror image peptides and proteins for therapeutic or synthetic biology applications.

## Graphical abstract

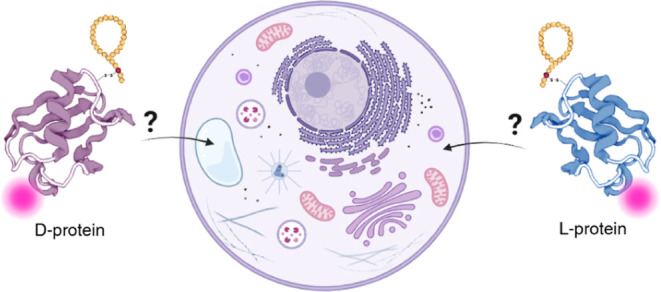

## Introduction

Mirror-image peptides and proteins made of D-amino acids are emerging as robust therapeutic scaffolds with unique advantages over their natural L-counterparts, including resistance to proteolytic degradation and low immunogenicity.^1,2^ Mirror-image phage display, the Random Nonstandard Peptides Integrated Discovery (RAPID) system, and computational protein design have further increased their usefulness by enabling the discovery of D-peptide and protein binders that target native D-proteins.^3–7^ Beyond these applications, mirror-image systems also intersect with fundamental questions about the origin of life and biological homochirality, probing why early evolution selected a single chiral form and whether alternative handedness could sustain life-like chemistry.^8–10^ In this context, significant progress toward the chemical synthesis of mirror-image biomolecules has provided conceptual and experimental foundations for exploring the assembly of entirely orthogonal mirror-image living systems.^11–14^ Because chemical synthesis is the only route to obtain these mirror-image biomolecules,^15–17^ their cellular utility ultimately depends on efficient intracellular delivery. This requirement raises a fundamental question: whether mirror-image cargoes enter cells with the same efficiency and via the exact mechanisms, or whether stereochemistry dictates distinct uptake behaviors (Figure 1).

**Figure 1:**
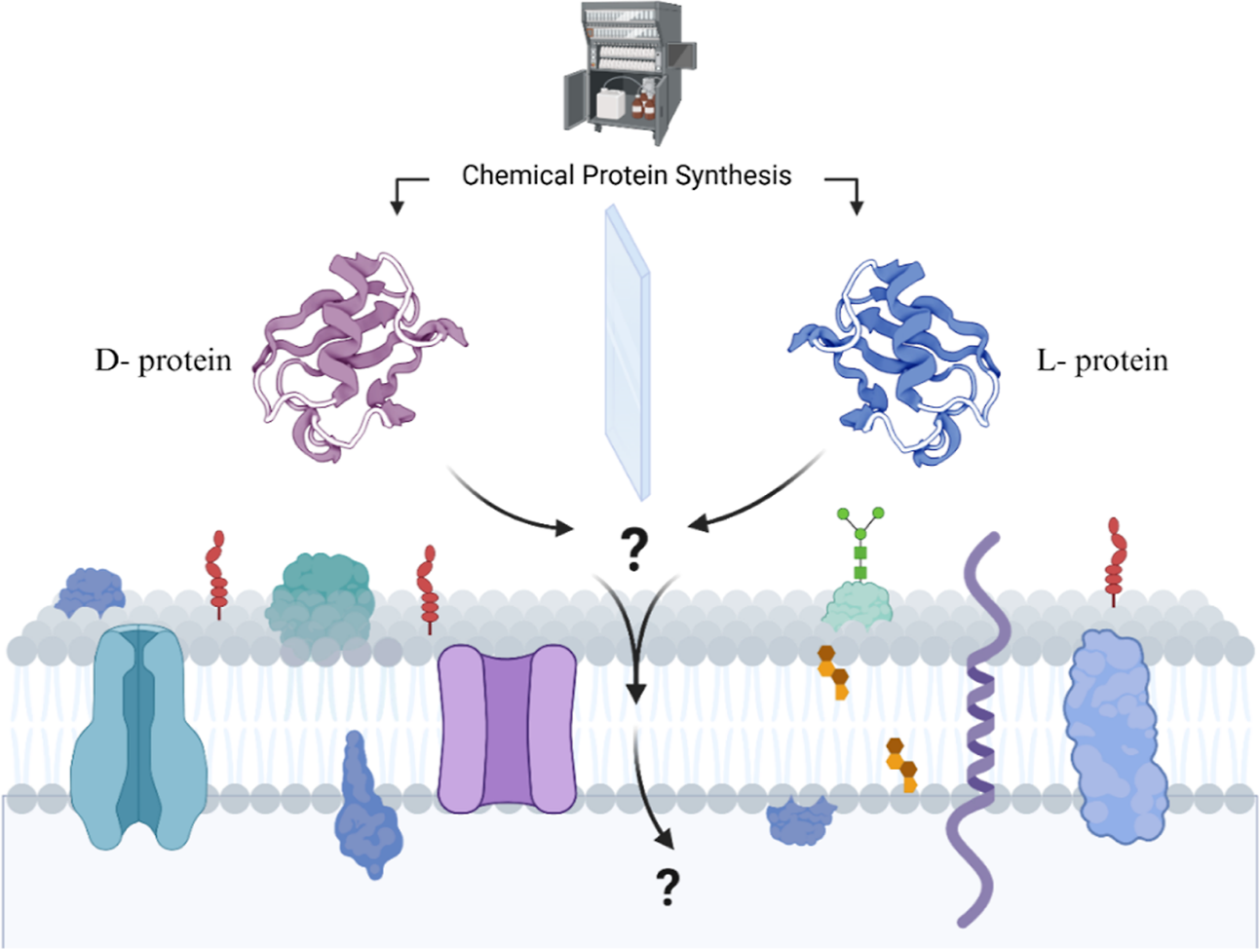
Chemical protein synthesis to probe cellular delivery of mirror-image proteins.

The plasma membrane offers a particularly compelling context for this question because of its profoundly homochiral assembly.^18^ Phospholipid headgroups adopt defined stereochemical configurations, membrane proteins are composed exclusively of L-amino acids, and cell–surface glycans incorporate D-sugar units.^19^ This asymmetric architecture endows the membrane with an intrinsically chiral interface, where recognition, binding, and transport are presumed to be enantioselective. Recent work by Craik and co-workers has shown that lipid chirality can modulate the deep insertion of membrane-active peptides, highlighting the membrane’s ability to discriminate between enantiomeric structures.^20^ However, this stereochemical sensitivity is restricted to membrane-active peptides and does not extend to biomolecules that interact with the cell surface and enter cells via alternative nonbilayer-penetrating pathways.

Cell-penetrating peptides (CPPs) provide a powerful platform for probing stereochemical effects by enabling transient cargo attachment, thereby isolating stereochemistry as the sole variable.^21–23^ Prior comparisons of L- and D-CPPs have revealed that chirality can influence their interactions with cell–surface components and uptake dynamics, with some studies reporting preferential uptake of L- or D-CPPs in a cell-type-dependent manner.^24^ For example, studies using the CPP penetratin (PEN) demonstrated that stereochemistry can affect both membrane association and the functional intracellular delivery of a conjugated L-cargo, such as insulin, indicating that enantiomeric inversion of the CPP can alter biologically relevant uptake behavior.^25^ In another study, Pentelute and co-workers evaluated chirality using the anthrax protective antigen (PA) system, attaching L- and D-polypeptide cargos to the L-configured N-terminal domain of lethal factor (LF_N_). PA first recognizes the L-form LF_N_, after which the pore unfolds and translocates appended chains independently of their chirality, delivering L- and D-cargos with comparable efficiency.^26^ While informative, this translocon-based mechanism (i.e., PA) cannot determine whether cargo stereochemistry affects uptake routes that operate without protein-assisted translocation and via other entry mechanisms.

These limitations highlight the need for a platform in which the cargo’s stereochemistry can be varied and reflected in the delivery efficacy, independent of the delivery vehicle and the mode of entry. CPPs uniquely provide this control, enabling cargos of defined chirality to be presented on an identical scaffold. More broadly, chemical protein synthesis and intracellular delivery strategies provide complementary tools for systematically interrogating synthetic protein function in cellular environments.^27–29^ Here, we address this gap by synthesizing three model cargos of increasing size and structural complexity in both L- and D-configurations, each conjugated to an identical cyclic deca-arginine (cR10) CPP.^30,31^ By quantifying intracellular uptake across multiple cell lines, we directly compared the uptake efficiency of L- and D-configured cargos under similar conditions. Our results reveal consistently higher uptake of L-cargos relative to their mirror image D-counterparts, demonstrating that cargo chirality is a key determinant of CPP-mediated uptake across the chiral biological membrane.

## Results and Discussion

To investigate how cargo chirality influences CPP-mediated intracellular delivery, we synthesized three pairs of enantiomeric cargos spanning a range of increasing structural complexity and size: the unstructured SpyTag peptide (ST), the de novo-designed structured mini-protein FW1 attached to ST, and the globular 76-residue ubiquitin (Ub) protein. ST is a short, intrinsically disordered peptide originally derived from the split SpyCatcher–SpyTag system, where it mediates spontaneous covalent isopeptide bond formation with its protein partner.^32^ This makes ST a suitable model for a minimally structured cargo dominated by primary sequence rather than stable tertiary interactions. By contrast, FW1 is a previously reported ∼40-residue mini-protein that adopts a defined fold composed of three β-strands and one α-helix.^33^ Attachment of FW1 to ST creates an intermediate cargo with defined secondary and tertiary structure, bridging the structural gap between the unstructured ST peptide and the fully globular Ub protein. Its intermediate size and structural organization position it between ST and Ub, allowing us to systematically examine how increasing structural complexity and size influence chirality-dependent uptake.

Following solid-phase peptide synthesis (SPPS) of all cargos in both L- and D-configurations, they were conjugated to an identical CPP scaffold: the cleavable cyclic deca-arginine peptide cR10 modified with 4-(dimethylaminoazo) benzene-4-carboxylic acid (DABCYL). Our group previously demonstrated that DABCYL and its derivatives enhance delivery efficiency by several-fold.^30,31^ A non-native cysteine residue was introduced in the C-termini of all cargo sequences to allow conjugation to the cR10DABCYL CPP via a disulfide bond. This disulfide linkage is designed to be cleaved intracellularly upon exposure to elevated cytosolic glutathione levels, thereby releasing the cargo.^34–37^ The additional two amino acids at the C-terminal of Ub prevent conjugation of the internalized Ub by the enzymatic machinery, thereby simplifying quantification by gel analysis. In addition, all constructs were labeled at the N-terminus with 5-carboxytetramethylrhodamine (TAMRA) to enable visualization and quantitative analysis of intracellular uptake. Notably, the use of identical CPPs and fluorophores across all constructs ensured that the only experimental variable in our study was the cargo’s chirality.

All six constructs—L-TAMRA-ST-CPP, D-TAMRA-ST-CPP, L-TAMRA-FW1-ST-CPP, D-TAMRA-FW1-ST-CPP, L-TAMRA-Ub-CPP, and D-TAMRA-Ub-CPP—were obtained in high purity and yield (complete synthetic procedures, purification details, and yield calculations for all intermediates and final compounds are provided in Supporting Information Sections 8–10), as confirmed by analytical HPLC and electrospray mass spectrometry (Figure 2A–C). Circular dichroism (CD) spectroscopy further confirmed the proper folding and stereochemical integrity. For each enantiomeric pair, the D construct exhibited a CD spectrum that was the mirror image of its L counterpart. The mirror-image spectra observed for **TAMRA-FW1-ST** and **TAMRA-Ub**, in the absence of the CPP, confirm that the constructs adopt the correct enantiomeric folds rather than misfolded or unfolded conformations (Figure 2B,C). In contrast, the random coil signature of **TAMRA-ST** (Figure 2A) is consistent with its intrinsically disordered nature.

**Figure 2:**
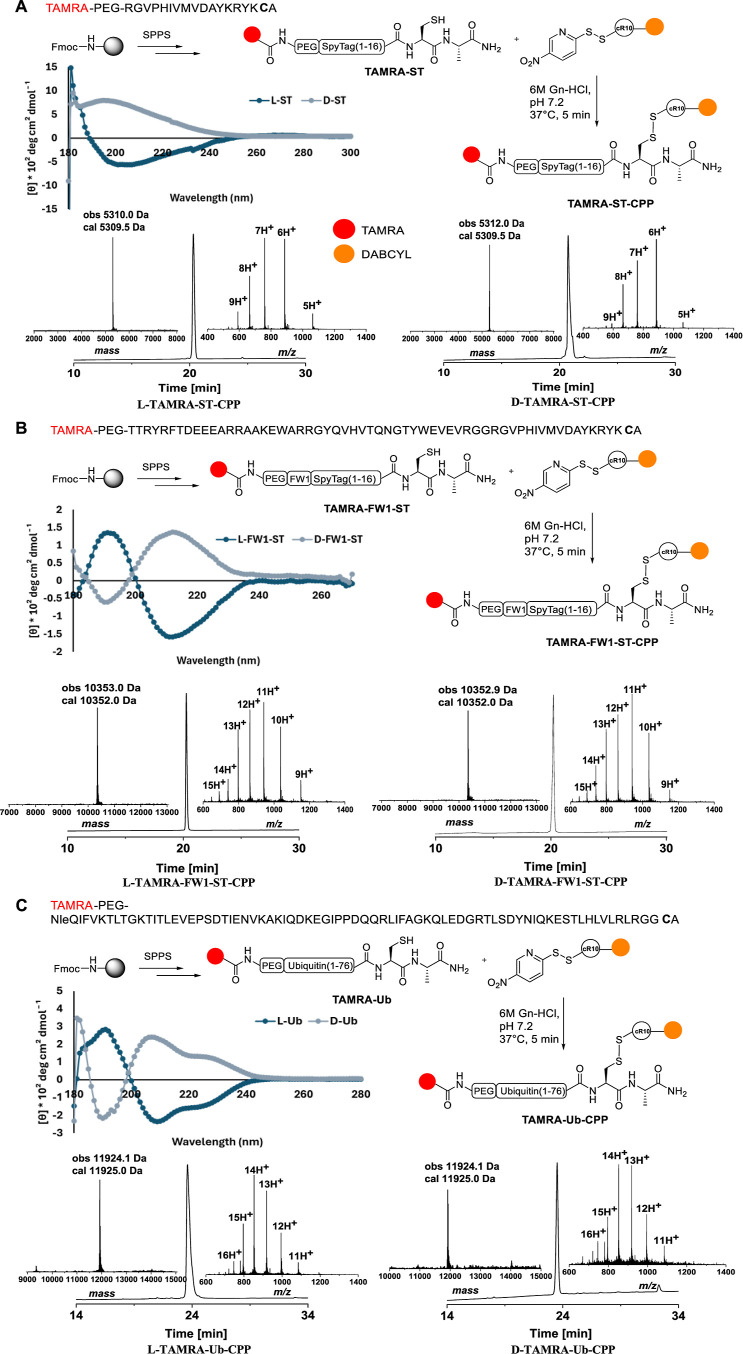
Synthesis and structural characterization of mirror-image cargos conjugated to cR10-DABCYL. (A) Synthesis scheme, CD spectra, analytical HPLC, and ESI-MS of purified **L-** and **D-TAMRA-ST-CPP** with the observed mass of 5310.0 ± 0.6 Da and 5312.0 ± 0.7 Da, respectively (calc. 5309.5 D). (B) Synthesis scheme, CD spectra, analytical HPLC, and ESI-MS of purified **L**- and **D-TAMRA-FW1-ST-CPP** with the observed mass of 10353.0 ± 1.0 Da and 10353.4 ± 1.0 Da, respectively (calc. 10352.0 Da). C) Synthesis scheme, CD spectra, analytical HPLC, and ESI-MS of purified **L-** and **D-TAMRA-Ub-CPP** with the observed mass 11924.1 ± 1.0 Da and 11924.3 ± 1.4, respectively (calc. 11925.0 Da).

With all six constructs in hand, we assessed whether TAMRA fluorescence quenched by DABCYL^38^ is restored upon GSH-mediated disulfide reduction. Our results from the fluorescence assay show an increased TAMRA signal upon GSH treatment, with comparable profiles for both L- and D-configurations, and at a level nearly identical to TAMRA-cargo without the CPP (representative fluorescence assay traces are shown in Figure S26 in the Supporting Information).

We next assessed how cargo stereochemistry affects CPP-mediated delivery by measuring cellular uptake (the total TAMRA signal of the cargo within the cytosol and that retained in the endosomes) using flow cytometry in the standard adherent cell lines HeLa and U2OS. L- and D-constructs of each cargo system were created and tested side by side at three concentrations (0.5, 1, and 2 μM) with an incubation period of 1 h, followed by extensive washing. The cellular uptake of all cargo systems, in both L- and D-forms, was measured by flow cytometry (Figure 3). Across all concentrations and cargo types, L-cargos consistently showed significantly higher intracellular fluorescence than their D-versions, with differences exceeding 1.8-fold in every case. Importantly, while the preference for L over D was seen in all three systems, the size of the difference in uptake decreased as cargo size increased: **L-TAMRA-ST** > **L-TAMRA-FW1-ST** > **L-TAMRA-Ub**, with fold differences of approximately 3, 2.2, and 1.8 in HeLa cells and approximately 4, 3, and 2.1 in U2OS cells, respectively (Figure 3A–C). Larger, more structured cargos, such as Ub, may allow stronger surface interactions with plasma membrane components; therefore, their internalization pathway might be less affected by stereochemistry. Additionally, U2OS cells consistently showed a bigger difference in uptake between L- and D-cargos than HeLa cells, which may reflect inherent differences in the chemical and physical properties of their plasma membranes. Indeed, it has been reported that U2OS cells have a thinner and less rigid actin cortex than HeLa cells,^39^ a property associated with enhanced membrane ruffling and macropinocytic activity.^40^

**Figure 3:**
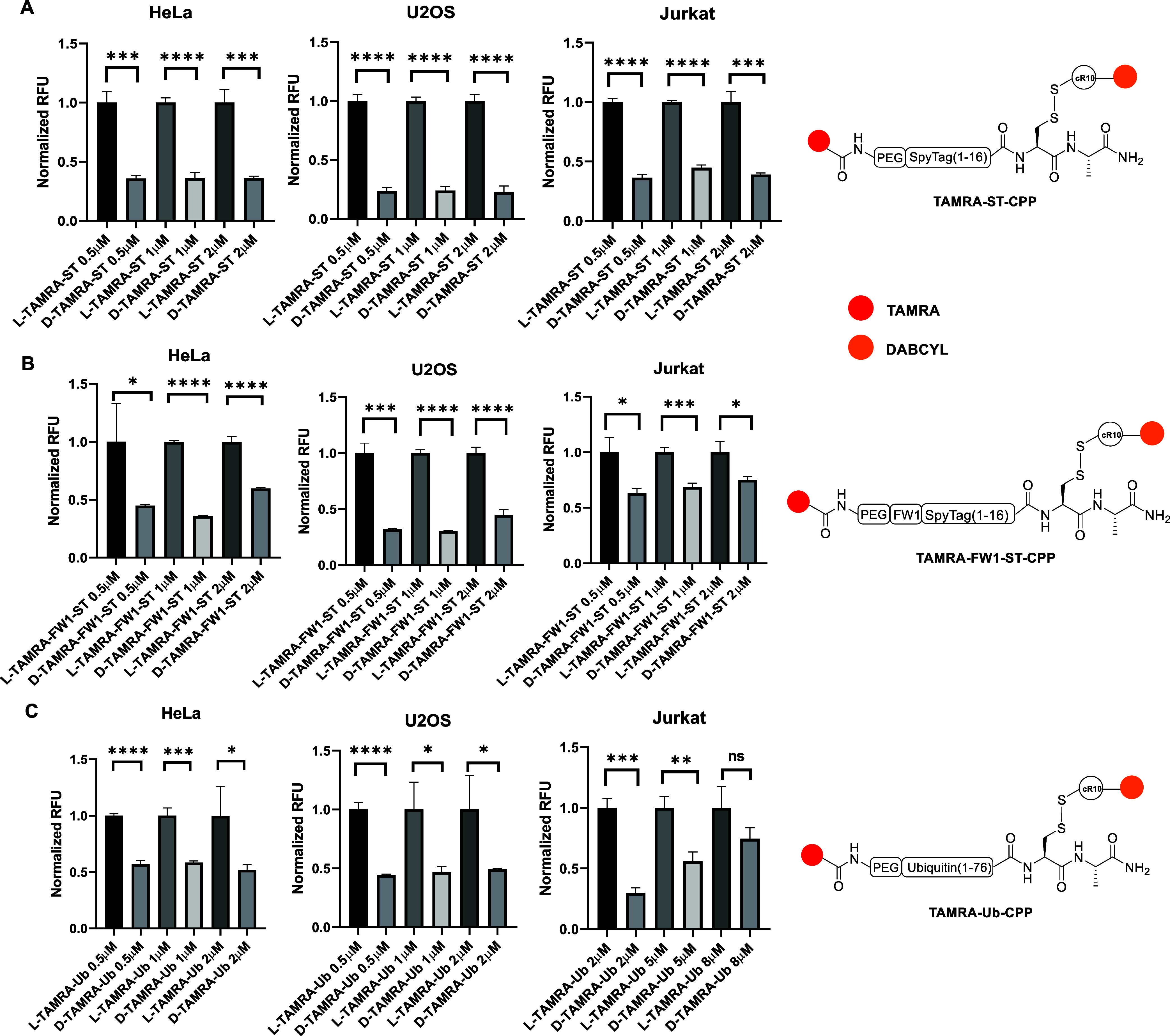
Flow cytometry analysis of TAMRA fluorescence intensities reports the cellular uptake of each cargo and its mirror-image analogue (linked to CPP) across HeLa, U2OS, and Jurkat cell lines at three different concentrations: 0.5 μM, 1 μM, and 2 μM following 1 h of incubation. (A) Quantification of uptake of **L**- and **D-TAMRA-ST-CPP**, (B) quantification of uptake of **L**- and **D-TAMRA-FW1-ST-CPP**, and (C) quantification of uptake of **L-** and **D-TAMRA-Ub-CPP**. Data are presented as mean values plus or minus SD for *n* = 3. Statistical significance was determined using an unpaired two-tailed *t*-test. Significance is indicated as ns = not significant (*p* ≥0.05), *p <0.05, **p <0.01, ***p <0.001, ****p <0.0001.

Heparan sulfate (HS) proteoglycans are abundant negatively charged glycans on the cell surface that serve as primary binding sites for many cationic CPPs.^41^ Interaction with HS chains facilitates initial electrostatic engagement of CPPs with the plasma membrane, concentrating the conjugates at the cell surface and often promoting subsequent internalization. Given their role in mediating CPP binding, variations in HS expression can influence the efficiency of uptake.^24,42^ To evaluate the role of HS-mediated interactions, uptake was measured in Jurkat cells, which lack HS proteoglycans on their surface. Previous reports have shown that in Jurkat cells, no differences in uptake were observed between L- and D-CPP.^24^ Whether this holds when changing the cargo’s stereochemistry rather than the CPP remains to be determined. As expected for HS-negative cells, the overall TAMRA fluorescence signal from each internalized cargo was lower than that of the other cell lines, consistent with their role in CPP-mediated cargo delivery (representative flow cytometry histograms comparing TAMRA fluorescence intensity across U2OS and Jurkat cells are provided in Figures S16–S18 of the Supporting Information).^42^ The reduction in uptake was particularly pronounced for larger cargos such as **TAMRA-Ub-CPP**, necessitating higher concentrations to maintain quantifiable signals. Interestingly, although the L/D difference in Jurkat cells was smaller than in HeLa and U2OS, L-cargo still exhibited higher uptake than D-cargo in the **ST** and **FW1-ST** system at all concentrations (0.5, 1, and 2 μM). For the Ub cargo system, **L-TAMRA-Ub** showed significantly higher uptake at 2 and 5 μM, whereas at 8 μM, the difference was no longer significant (Figure 3C). This decline in stereochemical preference at higher concentrations (>5 μM) might be attributed to an increased contribution of direct membrane translocation—a mechanism known to become more prominent at elevated CPP concentrations—which may diminish the difference in uptake between L- and D-cargos.^43^ Nonetheless, the persistence of the L > D preference in Jurkat cells in all cargo systems at lower concentrations, where uptake is predominantly mediated by endocytosis,^43^ suggests that cargo chirality influences internalization steps via interactions with other cell surface components and/or downstream of HS binding. This observation could be attributed to the inversion of the cargo stereochemistry, as the CPP scaffold remains constant.

To further support our flow cytometry results, laser scanning confocal microscopy (LSCM) was performed. All three cargo systems were delivered to U2OS cells at a final concentration of 5 μM and incubated at 37 °C for 1 h. Consistent with the flow cytometry data, cells treated with L-cargos exhibited stronger intracellular fluorescence than those treated with D-cargos (Figure 4).

**Figure 4:**
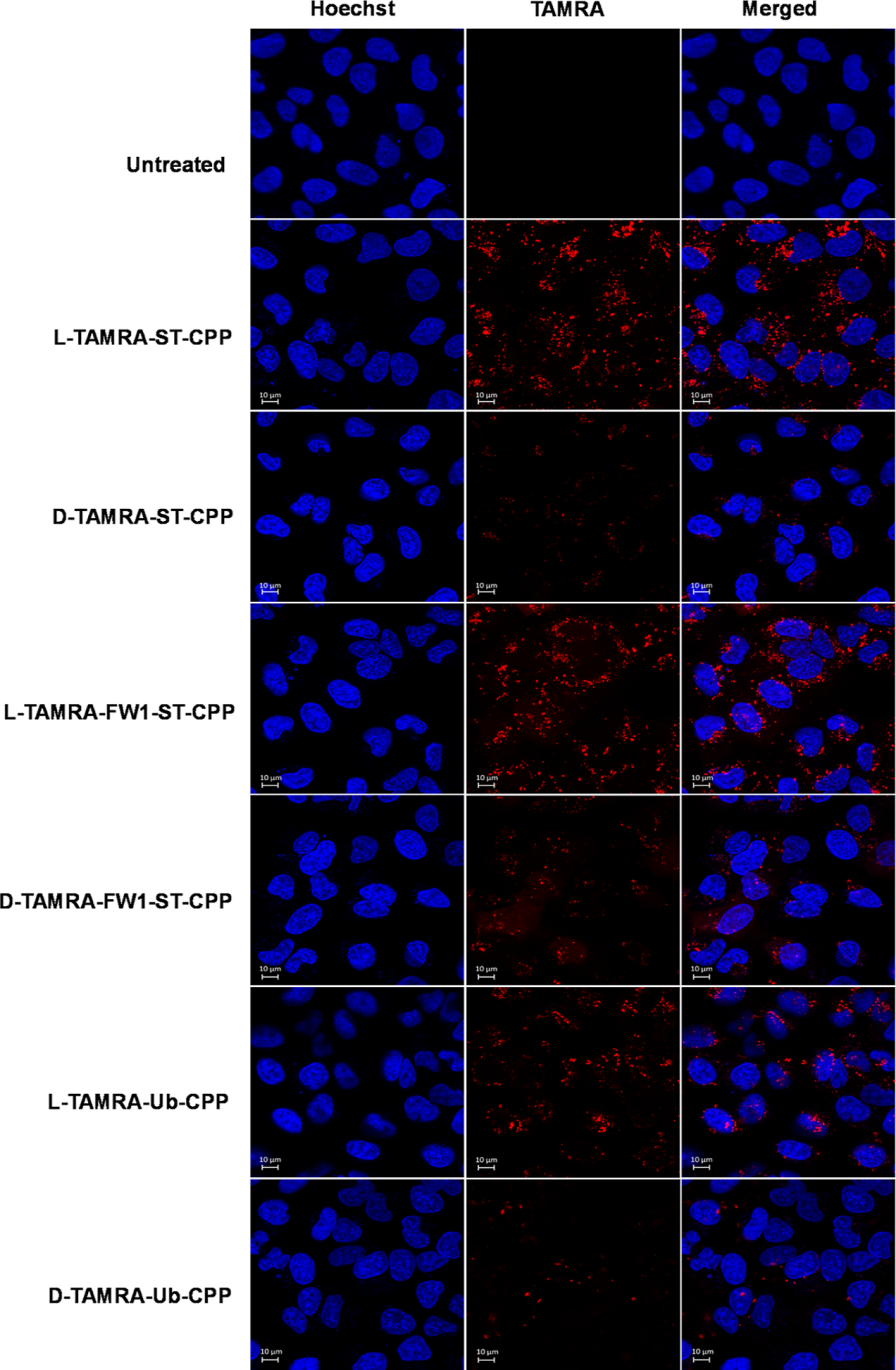
Live cell LSCM images (TAMRA channel) of U2OS cells after delivery of cargos and their mirror image forms at a concentration of 5 μM for 1 h. Hoechst was used as a nuclear stain (10 μm scale bar).

To enable quantitative assessment, *z*-stack images were acquired using a spinning-disk confocal microscope, enabling three-dimensional reconstruction and integrated signal analysis (representative spinning-disk confocal microscopy images illustrating the intracellular localization of the TAMRA-labeled cargos are provided in Figure S14 of the Supporting Information). Analysis of these data showed approximately a 2-fold difference in uptake between L- and D-cargos across all cargo systems.

To further validate intracellular cargo uptake, we performed SDS-PAGE analysis of cell lysates from HeLa, U2OS, and Jurkat cells treated with 5 μM **L-** and **D-TAMRA-Ub-CPP** for 1 h at 37 °C. Smaller constructs, **TAMRA-ST-CPP** and **TAMRA-FW1-ST-CPP**, fall below the practical detection limit of gel-based analysis and were therefore not included. SDS-PAGE, followed by TAMRA fluorescence detection, revealed that **L-TAMRA-Ub-CPP** accumulated approximately 3-fold more than **D-TAMRA-Ub-CPP** across all cell lines tested. This fold difference is higher than that observed by flow cytometry. Quantification of fluorescence intensity from two independent replicates (raw, uncropped SDS-PAGE and Western blot images corresponding to the quantified band intensities are provided in Figure S15 of the Supporting Information) using an unpaired two-tailed *t*-test confirmed the significance of this difference. The representative gel image is shown in Figure 5A, and the integrated intensity quantification is presented in Figure 5B.

**Figure 5:**
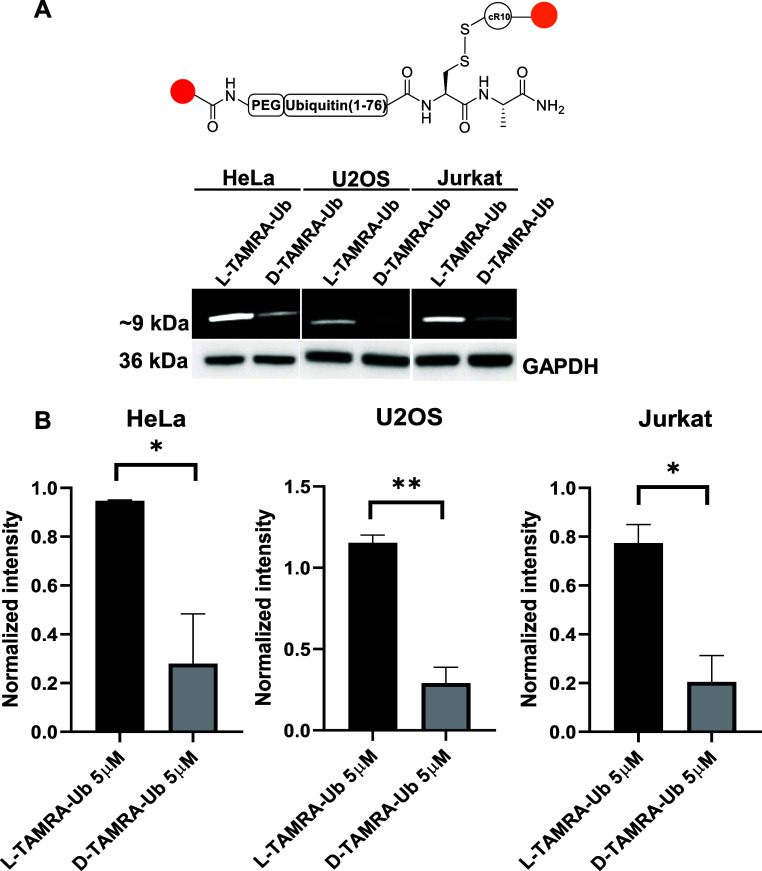
SDS PAGE analysis (TAMRA Channel) of intracellular delivery of **L-** and **D-TAMRA-Ub** following cell lysis. (A) SDS-PAGE analysis of **L-** and **D-TAMRA-Ub** uptake across three cell lines in the TAMRA channel. (B) Band intensity quantification of **L-** and **D-TAMRA-Ub** uptake across three cell lines. Data are presented as mean values ± SD, for *n* = 2. Significance is indicated as ns = not significant (*p* ≥0.05), *p <0.05, **p <0.01, ***p <0.001, ****p <0.0001.

It has been reported that cargo uptake mediated by CPPs can proceed through both energy-independent direct translocation and energy-dependent endocytic pathways, with the relative contribution of each route influenced by parameters such as concentration, the specific CPP sequence, cell type, and temperature.^43^ To determine whether the observed chiral differences in uptake depend on active cellular entry mechanisms, we examined CPP-cargo internalization under conditions that suppress endocytosis. Uptake was first assessed at 4 °C, a temperature known to markedly reduce energy-dependent processes, including receptor-mediated endocytosis.^44^ U2OS cells, which exhibited the most significant uptake under normal conditions, were treated with all cargo systems at a final concentration of 2 μM for 1 h, and TAMRA fluorescence was quantified by flow cytometry. Under these conditions, L-cargos showed uptake reductions exceeding 90%. In contrast, D-cargos exhibited a smaller decrease, with the effect most pronounced for **D-TAMRA-FW1-ST**, which displayed ∼60% reduction in uptake (Figure S16 in the Supporting Information shows quantitative analysis of fluorescence intensity reduction from 37 to 4 °C). At 4 °C, the pronounced L over D preference observed at 37 °C was largely abolished (Figure 6A). Specifically, no statistically significant differences were observed between **L-** and **D-TAMRA-ST-CPP** or between **L-** and **D-TAMRA-Ub-CPP**, indicating that the stereochemical bias observed at physiological temperature largely depends on active internalization. In contrast, **L-** and **D-TAMRA-FW1-ST-CPP** displayed an inverted preference at 4 °C, with the D-form exhibiting approximately 2-fold higher uptake than the L-form. This pattern suggests that the stereochemical bias observed at physiological temperature may, in part, arise from differences in interactions with chiral components of energy-dependent endocytic pathways. Consistent with the Jurkat experiments, these findings indicate that the cellular machinery involved in endocytic uptake could amplify chirality-dependent differences, rather than the bias being driven solely by initial surface interactions.

**Figure 6:**
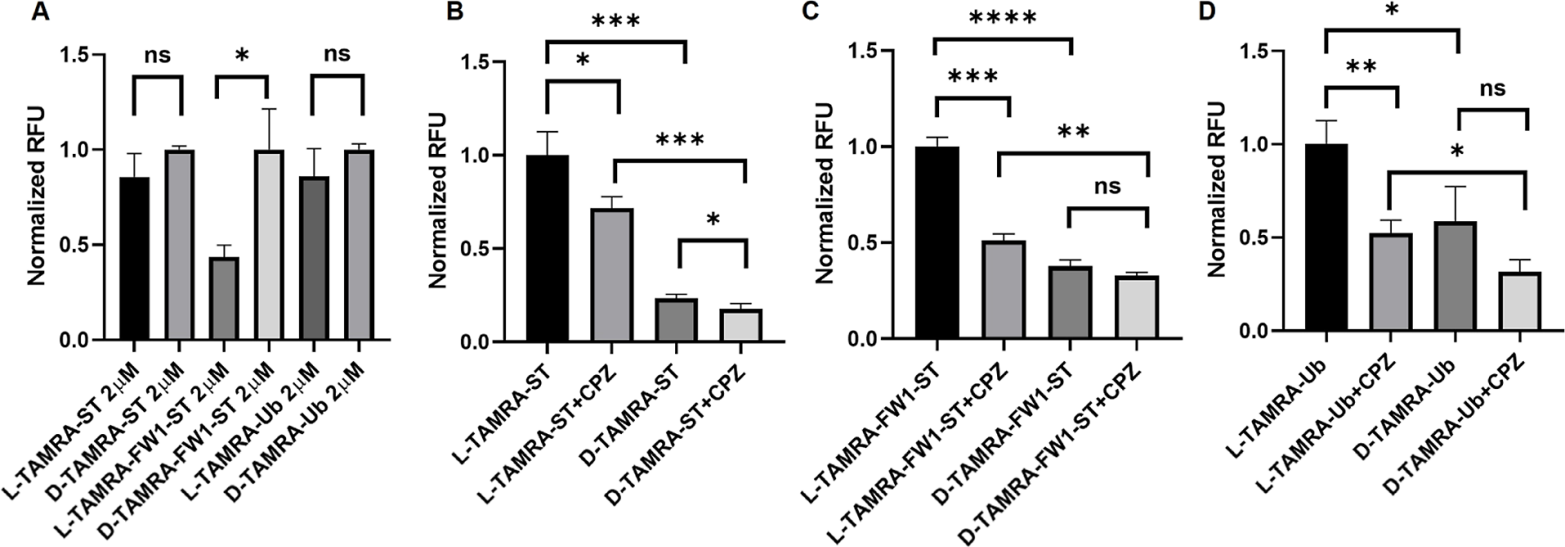
Flow cytometry analysis of TAMRA fluorescence intensity showing the cellular uptake of each cargo and its mirror-image form in U2OS cells under different conditions. (A) Quantification of uptake at 4 °C and (B) quantification of uptake of **L-** and **D-TAMRA-ST-CPP** following incubation with 20 μM of CPZ. (C) Quantification of uptake of **L-** and **D-TAMRA-FW1-ST-CPP** following incubation with 20 μM of CPZ. (D) Quantification of uptake of **L-** and **D-TAMRA-Ub-CPP** following incubation with 20 μM of CPZ. Data is presented as mean ± SD (*n* = 3). Significance is indicated as ns = not significant (*p* ≥0.05), *p <0.05, **p <0.01, ***p <0.001, ****p <0.0001.

To further probe the contribution of active endocytic pathways, we next assessed uptake in the presence of chlorpromazine (CPZ), which inhibits clathrin-mediated endocytosis by disrupting clathrin-coated pit formation by redistributing clathrin and its adaptor proteins from the plasma membrane to intracellular vesicles.^45^ Clathrin-mediated endocytosis is one of the mechanistic pathways that contribute to endocytic routes in mammalian cells and relies on highly organized protein assemblies that could, in principle, impose stereospecific interactions.^46^ U2OS cells were pretreated with 20 μM CPZ for 30 min, followed by treatment with all cargo systems at a final concentration of 2 μM for 1 h. Subsequently, TAMRA fluorescence intensities of the internalized cargos were quantified by flow cytometry. Notably, CPZ treatment reduced the overall uptake across all constructs, indicating that clathrin-dependent pathways contribute to their internalization. For ST cargos, uptake of the L-form decreased by 28% and the D-form by 24% in the presence of CPZ. Similarly, for Ub cargos, CPZ treatment reduced uptake of the L-form by 48% and the D-form by 46%. In both cases, the L-form exhibited a slightly greater reduction than the D-form. Under these conditions, the relative L/D difference was largely maintained, although modestly attenuated, indicating that clathrin-dependent internalization alone does not fully account for the stereochemical bias observed at physiological temperature (Figure 6B,D). In contrast, **L-TAMRA-FW1-ST-CPP** showed a ∼50% reduction, whereas the D-form was reduced by only ∼13% (Figure 6C). These results are consistent with the 4 °C experiment and indicate that clathrin-mediated pathways contribute disproportionately to L-form uptake. A similar trend was observed at 4 °C, where the L-form exhibited a marked reduction compared to the D-form (Figure 6A), consistent with greater reliance of **L-TAMRA-FW1-ST-CPP** on energy-dependent internalization mechanisms. Together, these findings support a model in which CPP-mediated delivery proceeds through multiple parallel uptake routes, with the relative contributions of clathrin-dependent and other energy-driven pathways varying according to cargo stereochemistry and structural features (i.e., size and sequence).

Having examined the contribution of clathrin-mediated endocytosis, we next assessed the contribution of macropinocytosis, another endocytic pathway commonly implicated in CPP-mediated cellular entry. To this end, U2OS cells were pretreated with the macropinocytosis inhibitor 5-(*N*-ethyl-*N*-isopropyl)­amiloride (EIPA) prior to cargo delivery. Notably, EIPA strongly reduced the uptake of both ST stereoisomers, with **L-TAMRA-ST-CPP** reduced by ∼85% and **D-TAMRA-ST-CPP** by ∼79%, suggesting that both forms rely substantially on macropinocytosis (flow cytometry analysis of EIPA-treated cells is provided in Supporting Information Figure S25). Together with their responses to CPZ, where both showed similar reduction in uptake, and to low temperature, these findings suggest that the higher uptake of **L-TAMRA-ST-CPP** at 37 °C reflects more efficient entry through the same uptake routes rather than differences in entry mechanisms.

The FW1-ST stereoisomers showed a moderate, nearly equivalent response to EIPA, with L-**TAMRA-FW1-ST-CPP** and **D-TAMRA-FW1-ST-CPP** reduced by ∼43% and ∼47%, respectively. The CPZ together with low-temperature experiment data suggest that the higher uptake of **L-TAMRA-FW1-ST-CPP** at 37 °C may be associated, in part, with greater engagement of a CPZ-sensitive, energy-dependent uptake component.

EIPA treatment reduced **L-TAMRA-Ub-CPP** and uptake by ∼83%, whereas **D-TAMRA-Ub-CPP** uptake decreased by ∼53%. Notably, this treatment abolished the uptake difference between the L- and D-cargos. Thus, the higher uptake of **L-TAMRA-Ub-CPP** at 37 °C and the greater reduction in the uptake of the L-isomer upon treatment with EIPA suggest greater dependence on macropinocytosis-associated uptake.

Together, these data suggest that the cargo chirality does not dictate a single uptake mechanism. Rather, stereochemistry appears to bias how each cargo engages a network of partially overlapping CPP-accessible pathways. The cellular membrane does not simply distinguish L- from D-cargos as isolated stereochemical objects but responds to chirality in the context of cargo identity, that is, size, structure, and composition.

To begin examining whether this stereochemical preference is restricted to the cR10 scaffold, we also evaluated a Penetratin (Pen)-based delivery system^23^ in U2OS cells. As shown in Supporting Information Figure S21, both **TAMRA-ST-Pen** and **TAMRA-FW1-ST-Pen** showed higher fluorescence for the L-cargo than for the corresponding D-cargo, with 3.3 and 1.3 fold increases, respectively. **TAMRA-Ub-Pen** was not included because of aggregation of the constructs under our experimental conditions. Although limited in scope, these preliminary data suggest that preferential L-cargo uptake may not be restricted to cR10 and that its magnitude likely depends on the cargo and CPP.

## Conclusions

In this study, we examined how cargo chirality influences cellular delivery, mediated by the same CPP scaffold. By directly comparing three mirror-image cargo pairs with increasing structural complexity, we demonstrate that L-cargoes consistently exhibit higher uptake than their D-counterparts across multiple cell lines, quantification methods, and mechanistic conditions. This bias is not solely due to differences in cell surface binding, as shown by its persistence in heparan sulfate-deficient cells, but instead results from differential engagement of active, energy-dependent internalization pathways. Notably, low-temperature and chlorpromazine or EIPA inhibition experiments indicate that this stereochemical preference arises primarily from endocytic uptake rather than from passive membrane interactions.

Our findings reveal that cargo chirality is an independent and robust determinant of the CPP-mediated uptake. The stereochemistry of the attached cargo reshapes the properties of the CPP-cargo ensemble, thereby influencing its engagement with the membrane, its induction of endocytic entry, and its accumulation within cells. This has important implications for the development of mirror-image peptide and protein therapeutics, which rely on D-amino acid scaffolds for enhanced stability but may face delivery disadvantages if stereochemical considerations are not addressed.

The cargo-chirality-dependent delivery in our system, which contrasts with that in the PA delivery approach,^26^ and the differences we observed in delivery when direct or endocytic mechanisms are used suggest that mirror-image synthetic proteins could also provide a unique platform for elucidating the mechanistic aspects of delivery pathways. Overall, this study offers a conceptual framework for understanding how chirality modulates CPP-mediated protein transport and delivery and underscores the need to account for cargo-dependent effects when designing delivery strategies for mirror image proteins.

The present study employs cyclic decarginine as a defined CPP scaffold to isolate the effect of cargo chirality under controlled conditions. The preliminary experiments with Penetratin provide an initial step toward testing CPP generality, but additional scaffolds,^47^ cargoes, and cell types will be required to define the scope of this effect. Work on intrinsically cell-permeable synthetic transcription factors^48,49^ illustrates how protein features can enable uptake, and examining mirror-image versions of such proteins could reveal whether stereochemistry influences uptake efficiency or entry mechanisms in the absence of CPPs. Similarly, grafting mirror-image proteins to cell-penetrating poly­(disulfides)^50^ could enable the exploration of how stereochemistry affects thiol-mediated, traceless delivery in the absence of classical CPPs. Moreover, coupling minimal fluorogenic labeling with synthetic L- and D-cargoes could enable real-time tracking to uncover potential stereospecific differences in cellular entry and trafficking.^51^

## Supplementary Material



## Notes

Experimental procedures, reagents, peptide and protein synthesis, HPLC and MS characterization, cell culture and cellular uptake protocols, flow cytometry analysis, confocal microscopy, SDS-PAGE and Western blot analysis, inhibition experiments, fluorescence assays, and supplementary figures (PDF)

## References

[1] Dintzis H. M., Symer D. E., Dintzis R. Z., Zawadzke L. E., Berg J. M. (1993). A Comparison
of the Immunogenicity of a Pair of Enantiomeric Proteins. Proteins.

[2] Milton R. C., Milton S. C., Kent S. B. (1992). Total chemical synthesis of a D-enzyme:
The enantiomers of HIV-1 protease show reciprocal chiral substrate
specificity. Science.

[3] Schumacher T. N. M., Mayr L. M., Minor D. L., Milhollen M. A., Burgess M. W., Kim P. S. (1996). Identification of d -Peptide Ligands
Through Mirror-Image Phage Display. Science.

[4] Welch B. D., VanDemark A. P., Heroux A., Hill C. P., Kay M. S. (2007). Potent
D-Peptide Inhibitors of HIV-1 Entry. Proc. Natl.
Acad. Sci. U. S. A..

[5] Zhang B. B., Harrison K., Zhong Y., Maxwell J. W. C., Ford D. J., Calvey L. P., So S. S., Peterson F. C., Volkman B. F., Stone M. J., Bhusal R. P., Kulkarni S. S., Payne R. J. (2024). Discovery
of Selective Cyclic D-Sulfopeptide Ligands of the Chemokine CCL22
via Mirror-Image mRNA Display with Genetic Reprogramming. J. Am. Chem. Soc..

[6] Ghareeb H., Yi Li C., Shenoy A., Rotenberg N., Shifman J. M., Katoh T., Sagi I., Suga H., Metanis N. (2025). Mirror-Image Random Nonstandard Peptides Integrated
Discovery (MI-RaPID) Technology Yields Highly Stable and Selective
Macrocyclic Peptide Inhibitors for Matrix Metallopeptidase 7. Angew. Chem., Int. Ed..

[7] Sun K., Li S., Zheng B., Zhu Y., Wang T., Liang M., Yao Y., Zhang K., Zhang J., Li H., Han D., Zheng J., Coventry B., Cao L., Baker D., Liu L., Lu P. (2024). Accurate de Novo Design of Heterochiral Protein–Protein
Interactions. Cell Res..

[8] Blackmond D. G. (2010). The Origin
of Biological Homochirality. Cold Spring Harb.
Perspect. Biol..

[9] Meledin R., Brik A. (2019). Mirroring Life’s
Building Blocks. Cell
Chem. Biol..

[10] Weinstock M. T., Jacobsen M. T., Kay M. S. (2014). Synthesis
and Folding of a Mirror-Image
Enzyme Reveals Ambidextrous Chaperone Activity. Proc. Natl. Acad. Sci. U. S. A..

[11] Weidmann J., Schnölzer M., Dawson P. E., Hoheisel J. D. (2019). Copying
Life: Synthesis
of an Enzymatically Active Mirror-Image DNA-Ligase Made of D-Amino
Acids. Cell Chem. Biol..

[12] Ling J., Fan C., Qin H., Wang M., Chen J., Wittung-Stafshede P., Zhu T. F. (2020). Mirror-Image 5S Ribonucleoprotein Complexes. Angew. Chem. Int. Ed..

[13] Zhao L., Lu W. (2014). Mirror Image Proteins. Curr. Opin. Chem. Biol..

[14] Zhang G., Zhu T. F. (2024). Mirror-Image Trypsin
Digestion and Sequencing of D-Proteins. Nat.
Chem..

[15] Becker C. F. W., Brik A., Dawson P., Hackenberger C. P. R. (2014). Chemical
Protein Synthesis. J. Pept. Sci..

[16] Kent S. B. H. (2019). Novel
Protein Science Enabled by Total Chemical Synthesis. Protein Sci. Publ. Protein Soc..

[17] Harrison K., Mackay A. S., Kambanis L., Maxwell J. W. C., Payne R. J. (2023). Synthesis
and Applications of Mirror-Image Proteins. Nat.
Rev. Chem..

[18] Wächtershäuser G. (2003). From Pre-Cells
to Eukarya – a Tale of Two Lipids. Mol.
Microbiol..

[19] Cooper, G. M. Structure of the Plasma Membrane. In The Cell: A Molecular Approach. 2nd ed.; Sinauer Associates, 2000. https://www.ncbi.nlm.nih.gov/books/NBK9898/.

[20] Henriques S. T., Peacock H., Benfield A. H., Wang C. K., Craik D. J. (2019). Is the
Mirror Image a True Reflection? Intrinsic Membrane Chirality Modulates
Peptide Binding. J. Am. Chem. Soc..

[21] Futaki S., Suzuki T., Ohashi W., Yagami T., Tanaka S., Ueda K., Sugiura Y. (2001). Arginine-Rich
Peptides. An Abundant
Source of Membrane-Permeable Peptides Having Potential as Carriers
for Intracellular Protein Delivery. J. Biol.
Chem..

[22] Frankel A. D., Pabo C. O. (1988). Cellular Uptake of the Tat Protein from Human Immunodeficiency
Virus. Cell.

[23] Derossi D., Joliot A. H., Chassaing G., Prochiantz A. (1994). The Third
Helix of the Antennapedia Homeodomain Translocates through Biological
Membranes. J. Biol. Chem..

[24] Verdurmen W. P. R., Bovee-Geurts P. H., Wadhwani P., Ulrich A. S., Hällbrink M., van Kuppevelt T. H., Brock R. (2011). Preferential Uptake
of L- versus D-Amino Acid Cell-Penetrating Peptides in a Cell Type-Dependent
Manner. Chem. Biol..

[25] Birch D., Sayers E. J., Christensen M. V., Jones A. T., Franzyk H., Nielsen H. M. (2023). Stereoisomer-Dependent
Membrane Association and Capacity
for Insulin Delivery Facilitated by Penetratin. Pharmaceutics.

[26] Rabideau A. E., Liao X., Pentelute B. L. (2015). Delivery
of Mirror Image Polypeptides
into Cells. Chem. Sci..

[27] Mann G., Sadhu P., Brik A. (2022). Synthetic Proteins behind the Plasma
Barrier: Molecular Spies. Acc. Chem. Res..

[28] Shkolnik D., Dey S., Hasan M., Matunis M. J., Brik A. (2024). Chemical Protein Synthesis
Combined with Protein Cell Delivery Reveals New Insights on the Maturation
Process of SUMO2. Chem. Sci..

[29] Mann G., Satish G., Sulkshane P., Mandal S., Glickman M. H., Brik A. (2021). Synthesis and Delivery of a Stable Phosphorylated Ubiquitin Probe
to Study Ubiquitin Conjugation in Mitophagy. Chem. Commun..

[30] Mandal S., Mann G., Satish G., Brik A. (2021). Enhanced Live-Cell
Delivery of Synthetic Proteins Assisted by Cell-Penetrating Peptides
Fused to DABCYL. Angew. Chem., Int. Ed..

[31] Saha A., Mandal S., Arafiles J. V. V., Gómez-González J., Hackenberger C. P. R., Brik A. (2022). Structure–Uptake Relationship
Study of DABCYL Derivatives Linked to Cyclic Cell-Penetrating Peptides
for Live-Cell Delivery of Synthetic Proteins. Angew. Chem., Int. Ed..

[32] Craenmehr F. W. B., Gräwe A., Veenbrink V. A., Bellan R., Merkx M., Dankers P. Y. W. (2025). Employing the SpyTag-SpyCatcher Reaction for the Modification
of Supramolecular Polymers with Functional Proteins. Bioconjugate Chem..

[33] Blanchard P. L., Knick B. J., Whelan S. A., Hackel B. J. (2023). Hyperstable Synthetic
Mini-Proteins as Effective Ligand Scaffolds. ACS Synth. Biol..

[34] Franz L., Schneider A. F. L., Hackenberger C. P. R. (2021). Targeted
Subcellular Protein Delivery
Using Cleavable Cyclic Cell-Penetrating Peptide-Conjugates. Methods Mol. Biol. Clifton NJ..

[35] Schneider A. F. L., Kithil M., Cardoso M. C., Lehmann M., Hackenberger C. P. R. (2021). Cellular
Uptake of Large Biomolecules Enabled by Cell-Surface-Reactive Cell-Penetrating
Peptide Additives. Nat. Chem..

[36] Nischan N., Herce H. D., Natale F., Bohlke N., Budisa N., Cardoso M. C., Hackenberger C. P. R. (2015). Covalent
Attachment of Cyclic TAT
Peptides to GFP Results in Protein Delivery into Live Cells with Immediate
Bioavailability. Angew. Chem., Int. Ed..

[37] Herce H. D., Schumacher D., Schneider A. F. L., Ludwig A. K., Mann F. A., Fillies M., Kasper M.-A., Reinke S., Krause E., Leonhardt H., Cardoso M. C., Hackenberger C. P. R. (2017). Cell-Permeable
Nanobodies for Targeted Immunolabelling and Antigen Manipulation in
Living Cells. Nat. Chem..

[38] Crisalli P., Kool E. T. (2011). Multi-Path Quenchers: Efficient Quenching of Common
Fluorophores. Bioconjugate Chem..

[39] Angert I., Karuka S. R., Mansky L. M., Mueller J. D. (2022). Partitioning of
Ribonucleoprotein Complexes from the Cellular Actin Cortex. Sci. Adv..

[40] Swanson J. A. (2008). Shaping
Cups into Phagosomes and Macropinosomes. Nat.
Rev. Mol. Cell Biol..

[41] Nakase I., Niwa M., Takeuchi T., Sonomura K., Kawabata N., Koike Y., Takehashi M., Tanaka S., Ueda K., Simpson J. C., Jones A. T., Sugiura Y., Futaki S. (2004). Cellular Uptake
of Arginine-Rich Peptides: Roles for Macropinocytosis and Actin Rearrangement. Mol. Ther..

[42] Belting M. (2003). Heparan Sulfate
Proteoglycan as a Plasma Membrane Carrier. Trends
Biochem. Sci..

[43] Ruseska I., Zimmer A. (2020). Internalization Mechanisms
of Cell-Penetrating Peptides. Beilstein J. Nanotechnol..

[44] Goldenthal K. L., Pastan I., Willingham M. C. (1984). Initial
Steps in Receptor-Mediated
Endocytosis. The Influence of Temperature on the Shape and Distribution
of Plasma Membrane Clathrin-Coated Pits in Cultured Mammalian Cells. Exp. Cell Res..

[45] Wang L. H., Rothberg K. G., Anderson R. G. (1993). Mis-Assembly of
Clathrin Lattices
on Endosomes Reveals a Regulatory Switch for Coated Pit Formation. J. Cell Biol..

[46] McMahon H. T., Boucrot E. (2011). Molecular Mechanism and Physiological Functions of
Clathrin-Mediated Endocytosis. Nat. Rev. Mol.
Cell Biol..

[47] Anjous R., P K., Saha A. (2026). Strategies to Improve
Intracellular Delivery of Arginine-Rich
Cell-Penetrating Peptides. J. Mater. Chem. B.

[48] Harel O., Nadal-Bufi F., Nithun R. V., Yao Y. M., Afek A., Vendrell M., Jbara M. (2025). Chemical Engineering of Transcription
Factors Uncovered Cell-Permeable μMax Modulators. J. Am. Chem. Soc..

[49] Kahler J. P., Ellenbroek B. D., Van Der Noord V. E., Van De Water B., Pomplun S. J. (2026). ReCHEMbinant Stapling Enhances Intracellular Delivery
and Bioactivity of Engineered Protein Inhibitors. Chem.

[50] Cognet M., Renno G., Coelho F., Sakai N., Matile S. (2026). Grafting Cell-Penetrating
Poly­(Disulfide)­s to Substrates of Interest: Dynamic Covalent Bioconjugation
for Traceless Delivery. Angew. Chem. Int. Ed..

[51] Nadal-Bufi F., Nithun R. V., De Moliner F., Lin X., Habiballah S., Jbara M., Vendrell M. (2025). Late-Stage Minimal
Labeling of Peptides
and Proteins for Real-Time Imaging of Cellular Trafficking. ACS Cent. Sci..

