# Supplementary material for: Chemical Synthesis of Mirror-Image Proteins Reveals Chirality-Dependent Cellular Uptake Mediated by a Cell-Penetrating Peptide
Source: J Am Chem Soc. 2026 Jun 17;148(31):33039–48. doi: 10.1021/jacs.6c03792 (PMC13474939; doi:10.1021/jacs.6c03792)
Supplement: Supplementary file 1 [file ja6c03792_si_001.pdf]

Supplementary Information

Chemical Synthesis of Mirror-Image Proteins Reveals Chirality-Dependent Cellular Uptake Mediated by a Cell-Penetrating Peptide

Eman Nassar, Ashok Donthoju, Mahdi Hasan and Ashraf Brik*

Schulich Faculty of Chemistry

Technion-Israel Institute of Technology

3200008 Haifa (Israel)

E-mail: abrik@technion.ac.il

Table of contents

1. General reagents....3
2. List of protected L-amino acids....3
3. List of protected D-amino acids....3
4. Cell culture reagents....4
5. General procedure for Fmoc-solid phase peptide synthesis (SPPS)....4
6. General procedure for peptide analysis and purification....5
7. General cell culture procedure....5
8. Synthesis of peptide and protein- cargos....6
   8.1 Synthesis of L-TAMRA-ST....6
   8.2 Synthesis of D-TAMRA-ST....7
   8.3 Synthesis of L-TAMRA-FW1-ST....7
   8.4 Synthesis of D-TAMRA-FW1-ST....8
   8.5 Synthesis of L-TAMRA-Ub....9
   8.6 Synthesis of D-TAMRA-Ub....9
9. Synthesis of cR10DABCYL CPP....10
10. Conjugation reactions between cargos and CPP....11
   10.1 Synthesis of L-TAMRA-ST-CPP....11
   10.2 Synthesis of D-TAMRA-ST-CPP....12
   10.3 Synthesis of L-TAMRA-FW1-ST-CPP....12
   10.4 Synthesis of D-TAMRA-FW1-ST-CPP....13
   10.5 Synthesis of L-TAMRA-Ub-CPP....13
   10.6 Synthesis of D-TAMRA-Ub-CPP....14
11. Synthesis of Penetratin (Pen) CPP....14
12. Conjugation reactions between cargos and Pen CPP....15
12.1 Synthesis of L-TAMRA-ST-Pen....15
12.2 Synthesis of D-TAMRA-ST-Pen....16
12.3 Synthesis of L-TAMRA-FW1-ST-Pen....16
12.4 Synthesis of D-TAMRA-FW1-ST-Pen....17
12.5 Synthesis of D-TAMRA-Ub-Pen....18
13. Circular dichroism (CD) measurement....18
14. General procedure for cellular uptake studies using flow cytometry....18

15. General method for cell imaging using laser scanning and spinning-disk confocal microscopy and image analysis 20
16. Lysis protocol using RIPA buffer 23
17. Fluorescence SDS-PAGE gel analysis and western blot 23
18. General procedure for cargo uptake studies under 4 ℃ condition 24
19. General procedure for cargo uptake studies with endocytosis inhibitor, chlorpromazine 25
20. General procedure for cargo uptake studies with mmacropinocytosis inhibitor, 5-(N-Ethyl-N-isopropyl)amiloride (EIPA) 25
21. Fluorescence characterization of TAMRA-ST and Ub conjugates and GSH Reduction Assay........ 26

1. General reagents:

Resins were purchased from CreoSalus. Peptides were synthesized by SPPS either manually in Teflon filter-fitted syringes (purchased from Torviq) or by using an automated peptide synthesizer (CS336X, CSBIO). Reagents Tetrakis(triphenylphosphine)palladium(0) was purchased from Chem-Impex ($Pd(PPh_3)_4$), Phenylsilane ($PhSiH_2$), and 5,5'-Dithiobis(2-nitrobenzoic acid) (DTNP) were purchased from Thermo Scientific, 4-((4-(dimethylamino)phenyl)azo)benzoic Acid (Dabcyl) was purchased from FluroChem, 2-(3,6-Bis(dimethylamino)xanthylium-9-yl)-5-carboxybenzoate (TAMRA) and [2-[2-(9H-Fluoren-9-ylmethoxycarbonylamino)ethoxy]ethoxy]acetic acid (PEG) were purchased from Aaron chemicals. Activating reagents *N*,*N*,*N′*,*N′*Tetramethyl-O-(1H-benzotriazol-1-yl) uroniumhexafluorophosphate (HBTU), 1-Hydroxybenzotriazolemonohydrate(HOBt), O-(1H-6-Chlorobenzotriazole-1-yl)-1,1,3,3- tetramethyluronium hexafluorophosphate (HCTU), 1-[Bis(dimethylamino)methylene]-1H-1,2,3-triazolo[4,5-b]pyridinium 3-oxid hexafluorophosphate (HATU) were purchased from LuxembourgBio Technologies and 7-Azabenzotriazol-1-yloxy)tripyrrolidinophosphonium hexafluorophosphate (PyAOP) was procured from Novabiochem®. All solvents: N,N-diisopropylethylamine (DIEA), dichloromethane (DCM), N,N- dimethylformamide (DMF), Piperidine (Pip), acetonitrile (CAN), Diethyl ether (Et_2O) and Trifluoroacetic acid (TFA) were purchased from Bio-Lab. Additional miscellaneous chemicals were purchased from Merck, Strem chemicals, and Alfa Aesar. L-and D-amino acids were purchased from Aaron Chemicals.

2. List of protected L-amino acids:

Fmoc-Gly-OH, Fmoc-Ala-OH, Fmoc Val-OH, Fmoc-Leu-OH, Fmoc-Ile-OH, Fmoc-Phe-OH, Fmoc-Pro-OH, Fmoc-His(Trt) OH, Fmoc-Asn(Trt)-OH, Fmoc-Gln(Trt)-OH, Fmoc-Arg(Pbf)-OH, Fmoc-Lys(Boc)-OH, Fmoc-Tyr(tBu)-OH, Fmoc-Ser(tBu)-OH, Fmoc-Thr(tBu)-OH, Fmoc-Asp(OtBu)-OH, Fmoc-Glu(OtBu)-OH, Fmoc-Trp(Boc)-OH, Fmoc-Nle-OH, Fmoc-Cys(Acm)-OH, Fmoc Cys(Trt)-OH, Boc-Val-OH, Fmoc-(Dmb)Gly-OH, Fmoc-Lys(Alloc)-OH, Fmoc-Lys(Dde) OH, Fmoc-Glu(OAll)-OH, Boc-Cys-OH, Fmoc-Met-OH.

3. List of protected D-amino acids:

Fmoc-Gly-OH, Fmoc-D-Ala-OH, Fmoc-D-Val-OH, Fmoc-D-Leu-OH, Fmoc-D-Ile-OH, Fmoc-D-Phe-OH, Fmoc-D-Pro-OH, Fmoc-D-His(Trt)-OH, Fmoc-D-Asn(Trt)-OH, Fmoc-D-Gln(Trt)-OH, Fmoc-D-Arg(Pbf) OH, Fmoc-D-Lys(Boc)-OH, Fmoc-D-Tyr(tBu)-OH, Fmoc-D-Ser(tBu)-OH, Fmoc-D Thr(tBu)-OH, Fmoc-D-Asp(OtBu)-OH, Fmoc-D-Glu(OtBu)-OH,

Fmoc-D-Trp(Boc)-OH, Fmoc-D-Nle-OH, Fmoc-D-Cys(Trt)-OH, Boc-D-Val-OH, Fmoc-(Dmb)Gly-OH, Fmoc-D-Met-OH.

4. Cell culture reagents:

Dulbecco's modified eagle's medium (DMEM), phenol free DMEM (Opti-DMEM), Roswell Park Memorial Institute medium (RPMI-1640), Fetal Bovine Serum (FBS), antibiotics (pencillin/streptomycin), Trypsin/EDTA, and phosphate-buffered saline (PBS) were purchased from Sigma Aldrich. Trans-blot turbo (0.2 µm PVDF) membranes for blotting and electrophoresis were purchased from Bio-Rad. Protease Inhibitor Cocktail, Hoechst 33342 solution (20 mM), and cell culture plates were purchased from Thermo-fischer. A non-protein Instant Block buffer for western blotting application was purchased from Gene Bio-Application. MOPS SDS running buffer powder, mPAGE™ 4-20% Bis-Tris Precast Gel, and Immobilon Crescendo Western HRP substrate were purchased form Millipore. µ-Slide 8 well for confocal microscopy was purchased from Ibidi, and poly-L-lysine hydrobromide was purchased form Sigma. [3-(2-chloro-10H-phenothiazin-10-yl)propyl]dimethylamine (chlorpromazine) was purchased from AA Blocks.

5. General procedure for Fmoc-solid phase peptide synthesis (SPPS):

Fmoc-based solid-phase peptide synthesis (Fmoc-SPPS) was performed either on an automated peptide synthesizer (C6336X, CSBIO) or manually using syringes fitted with Teflon filters. Rink amide resin was swollen for 1 h in a 1:1 (v/v) mixture of DCM and DMF. Fmoc deprotection was carried out using 20% piperidine in DMF for 2 × 5 min, followed by washing with DMF (3 × 10 mL). Amino acid coupling was performed using Fmoc-protected amino acids (4 eq.) activated with HCTU (4 eq.) and DIEA (8 eq.) relative to the resin loading, with a coupling time of 1 h. Following synthesis of the complete sequence, the resin was washed with DCM and dried under vacuum. To cleave the peptide from the solid support, dried resin was treated with 10-fold volume of cleavage cocktail containing TFA/water/triisopropylsilane (TIS) (95:2.5:2.5) and was shaken for 3 h at room temperature. Peptides were precipitated with 10-fold volume of cold diethyl ether and centrifuged at 4000 rpm for 15 mins. The precipitate was resuspended in water/acetronitrile and freeze-dried in the lyophilizer to obtain the crude peptide.

6. General procedure for peptide analysis and purification:

All synthesized peptides were purified high performance liquid chromatography (HPLC) and characterized by mass spectrometry. Analytical HPLC was performed on a reverse- phase Thermo instrument (Dionex Ultimate 3000) using Xbridge® (4.6 x 150 mm, 3.5 µm, 300 Å, BEH C4, waters) and XSelect™ (4.6 x 150 mm, 3.5 µm, 130 Å, CSH C18, waters) analytical columns with 1.2 mL/min flow rate. Semi-preparative HPLC was carried out on Thermo instrument (Dionex Ultimate 3000) using Xbridge® (10 x 250 mm, 5 µm, 300 Å, BEH C4, waters) and XSelect™ (10 x 150 mm, 5 µm, 130 Å, CSH C18, waters) semi-preparative columns with 4 mL/min flow rate. Preparative HPLC was performed on Xbridge® (19 x 250 mm, 5 µm, 300 Å, BEH C4, waters) and XSelect™ (19 x 250 mm, 5 µm, 130 Å, CSH C18, waters) analytical columns with 15 mL/min flow rate. Solvent A; water with 0.05% TFA and solvent B; acetonitrile with 0.05% TFA were used to as HPLC mobile phases. LCQ Fleet mas spectrometer (Thermo Scientific) with an ESI source was used to perform electrospray ionization mass spectrometry (ESI-MS).

7. General cell culture procedure:

HeLa (CCL-2™, ATCC) and U2OS (HTB-96™ ATCC) cells were cultured in high-glucose and low-glucose DMEM medium, respectively, supplemented with 10% FBS, 0.2 mM L-Gln and antibiotics (penicillin/streptomycin) (complete medium) at 37 ℃ in a humidified incubator with 5% CO_2. For passaging, culture medium was aspirated, and cells were washed with sterile calcium- and magnesium- free PBS, followed by incubation with 0.25% trypsin and 0.02% EDTA for 5 min at 37 ℃. Trypsinization was quenched by the addition of 2-fold volume of complete medium, and cells were then passaged and reseeded in fresh complete medium. Jurkat (TIB-152™, ATCC) cells were cultured in RPMI-1640 medium supplemented with 10% FBS, 0.2 Mm L-Gln and penicillin—streptomycin at 37 ℃ in a humidified incubator with 5% CO_2. Cells were maintained in suspension and kept in the exponential growth phase by passaging every 2-3 days, during which cultures were diluted with fresh complete medium to the desired cell density.

For flow cytometry analysis, cells suspensions were collected and pelleted (5 min at 500 x g for HeLa and U2OS cells, and 5 min at 200 x g for Jurkat cells). The supernatant was then aspirated, and the cell pellet was resuspended in fresh complete medium for HeLa and U2OS, or in serum-free medium for Jurkat cells. Cell density was determined by using and automated cell counter (Countess II, Invitrogen). Subsequently, cells were seeded in 12- or 24-well plates

at a density of 1 × 10⁶ cells per well for HeLa and U2OS, and 5 × 10⁵ cells per well for Jurkat cells and allowed to reach approximately 90% confluency prior to experiments. For confocal microscopy, HeLa and U2OS cells were seeded on poly-L-lysine–coated Ibidi 8-well chamber slides at 5 × 10⁴ cells per well and cultured for 24 h to reach approximately 90% confluency.

8. Synthesis of peptide and protein- cargos:

8.1 Synthesis of L-TAMRA-ST:

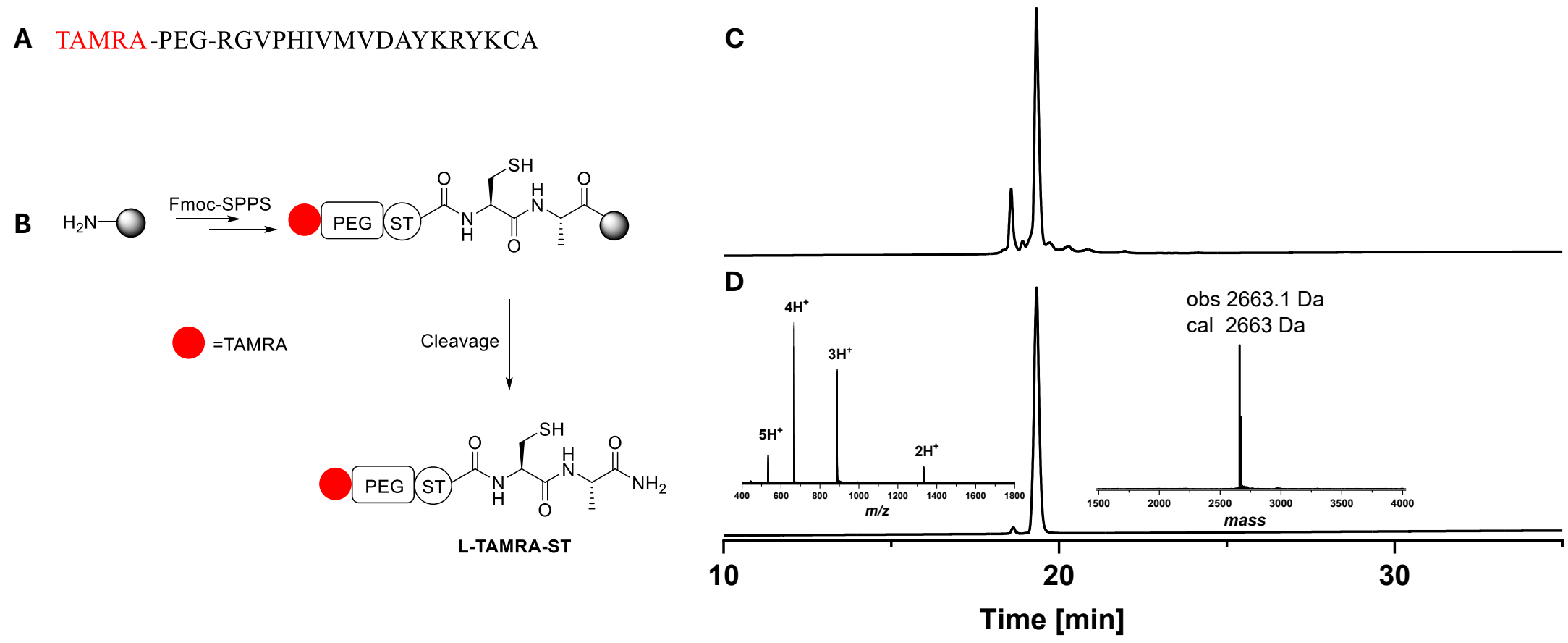

Figure S1. Synthesis of L-TAMRA-ST. **A**) Sequence of L-TAMRA-ST. **B**) Schematic representation of synthetic route. **C**) HPLC of crude L-TAMRA-ST. **D)** HPLC and mass analysis of purified L-TAMRA-ST with the observed mass 2663.1 ± 0.4 Da (calcd. 2663.5 Da, average isotopes).

L-TAMRA-ST was synthesized on rink amide resin (0.2 mmol) following the described general procedure of Fmoc-SPPS. The crude peptide was purified by preparative HPLC using a C18 column with a gradient of 5-65% B over 40 mins to afford the purified peptide in 23% yield.

8.2 Synthesis of D-TAMRA-ST:

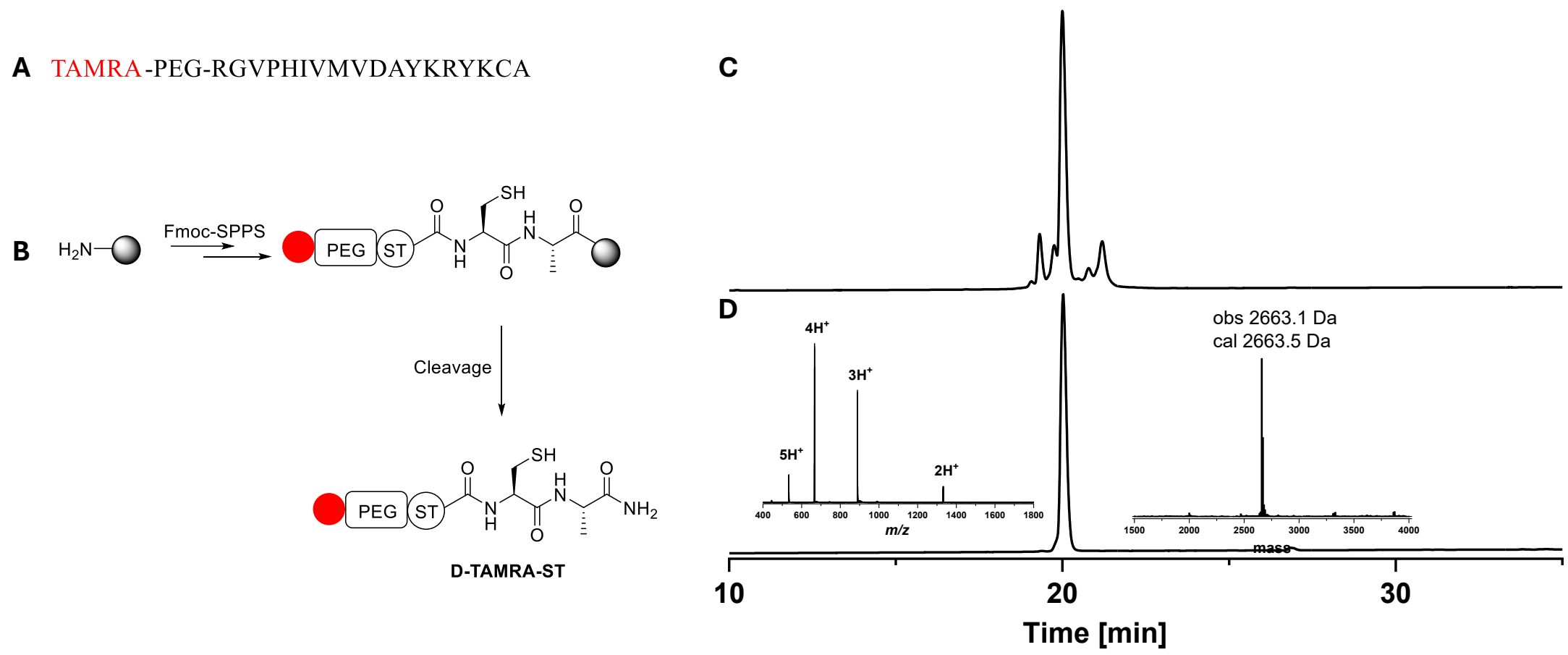

Figure S2. Synthesis of D-TAMRA-ST. **A**) Sequence of D-TAMRA-ST. **B**) Schematic representation of synthetic route. **C**) HPLC of crude D-TAMRA-ST. **D)** HPLC and mass analysis of purified D-TAMRA-ST with the observed mass 2664.7 ± 0.5 Da (calcd. 2663.5 Da, average isotopes).

D-TAMRA-ST was synthesized on rink amide resin (0.2 mmol) following the described general procedure of Fmoc-SPPS. The crude peptide was purified by preparative HPLC using a C18 column with a gradient of 5-65% B over 40 mins to afford the purified peptide in 21% yield.

8.3 Synthesis of L-TAMRA-FW1-ST:

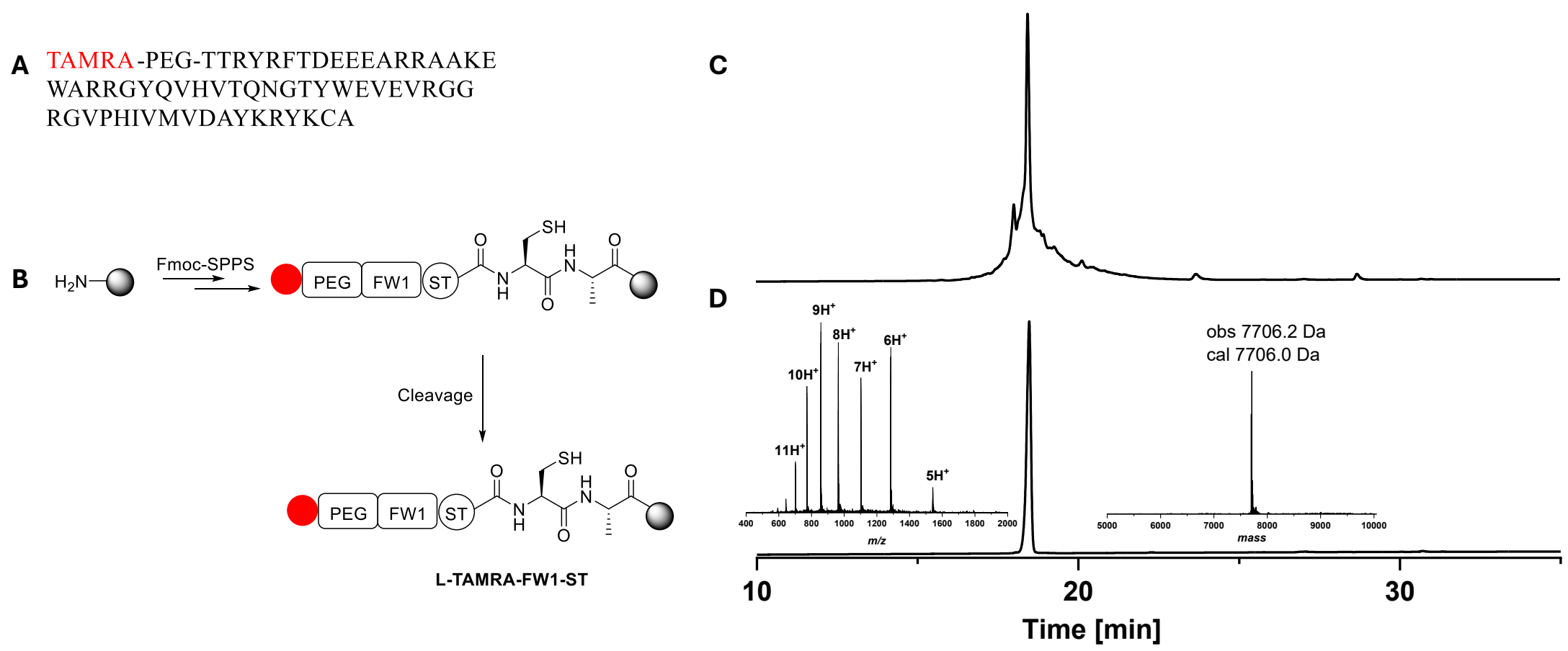

Figure S3. Synthesis of L-TAMRA-FW1-ST. **A**) Sequence of L-TAMRA-FW1-ST. **B**) Schematic representation of synthetic route for L-TAMRA-FW1-ST. **C**) HPLC of crude L-TAMRA-FW1-ST.

D) HPLC and mass analysis of purified L-TAMRA-FW1-ST with the observed mass 7706.2 ± 0.7 Da (calcd. 7706.0 Da, average isotopes).

L-TAMRA-FW1-ST was synthesized on rink amide resin (0.2 mmol) following the described general procedure of Fmoc-SPPS. The crude peptide was purified by preparative HPLC using a C44 column with a gradient of 5-65% B over 40 mins to afford the purified peptide in 18% yield.

8.4 Synthesis of D-TAMRA-FW1-ST:

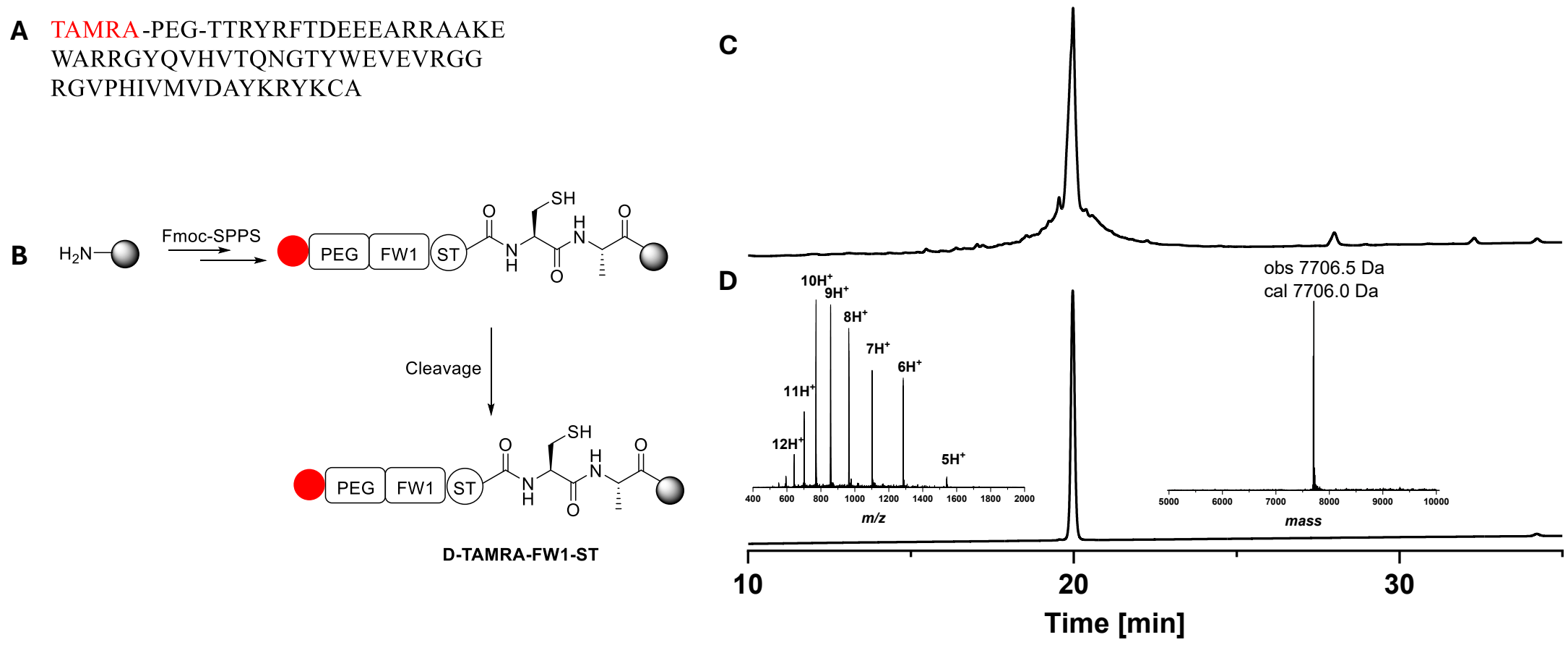

Figure S4. Synthesis of D-TAMRA-FW1-ST. **A**) Sequence of D-TAMRA-FW1-ST. **B**) Schematic representation of synthetic route for D-TAMRA-FW1-ST. **C**) HPLC of crude D-TAMRA-FW1-ST. **D)** HPLC and mass analysis of purified D-TAMRA-FW1-ST with the observed mass 7706.5 ± 0.8 Da (calcd. 7706.0 Da, average isotopes).

D-TAMRA-FW1-ST was synthesized on rink amide resin (0.2 mmol) following the described general procedure of Fmoc-SPPS. The crude peptide was purified by preparative HPLC using a C4 column with a gradient of 5-65% B over 40 mins to afford the purified peptide in 16% yield.

8.5 Synthesis of L-TAMRA-Ub:

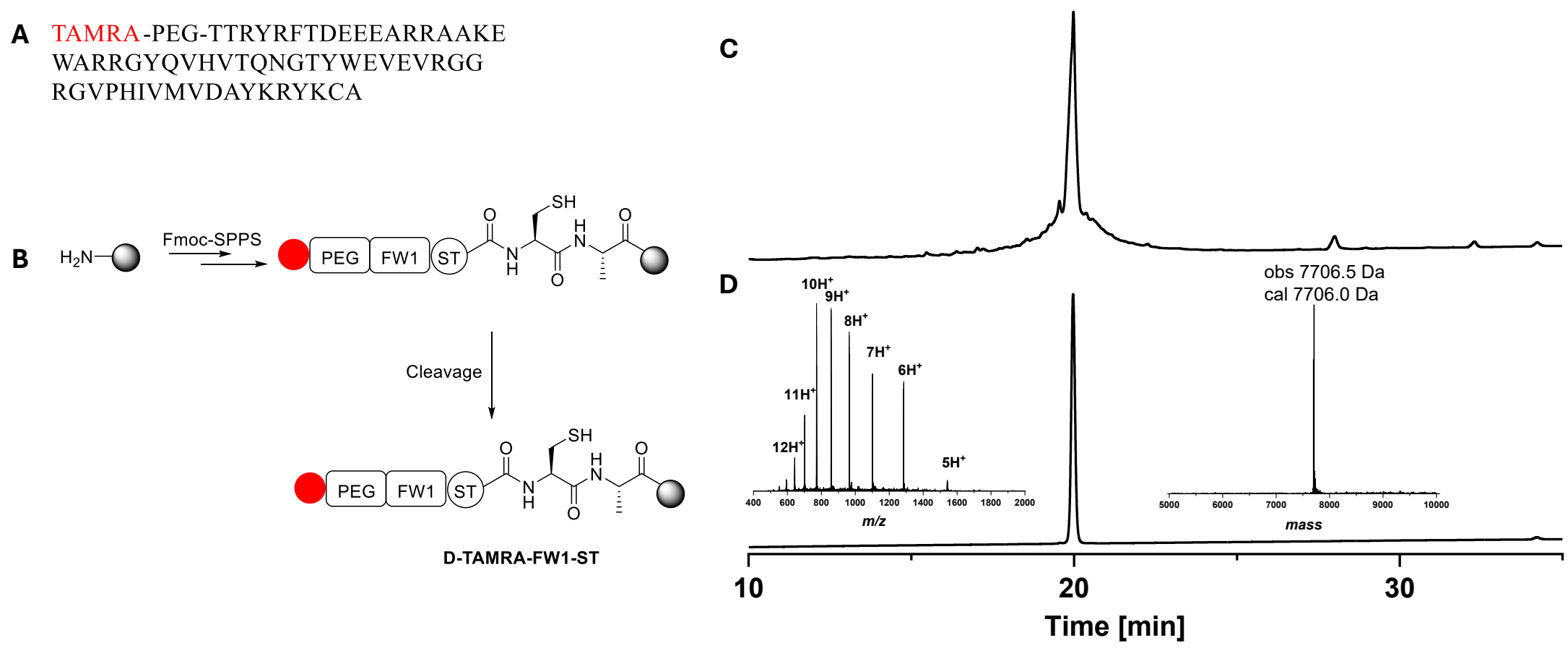

Figure S5. Synthesis of L-TAMRA-Ub (5) **A**) Sequence of L-TAMRA-Ub. (5) **B**) Schematic representation of synthetic route for L-TAMRA-Ub. **C**) HPLC of crude L-TAMRA-Ub. **D)** HPLC and mass analysis of purified L-TAMRA-Ub with the observed mass 9277.4 ± 0.8 Da (calcd. 9278.0 Da, average isotopes).

L-TAMRA-Ub was synthesized on rink amide resin (0.2 mmol) following the described general procedure of Fmoc-SPPS. The crude peptide was purified by preparative HPLC using a C18 column with a gradient of 5-65% B over 40 mins to afford the purified peptide in 17% yield.

8.6 Synthesis of D-TAMRA-Ub:

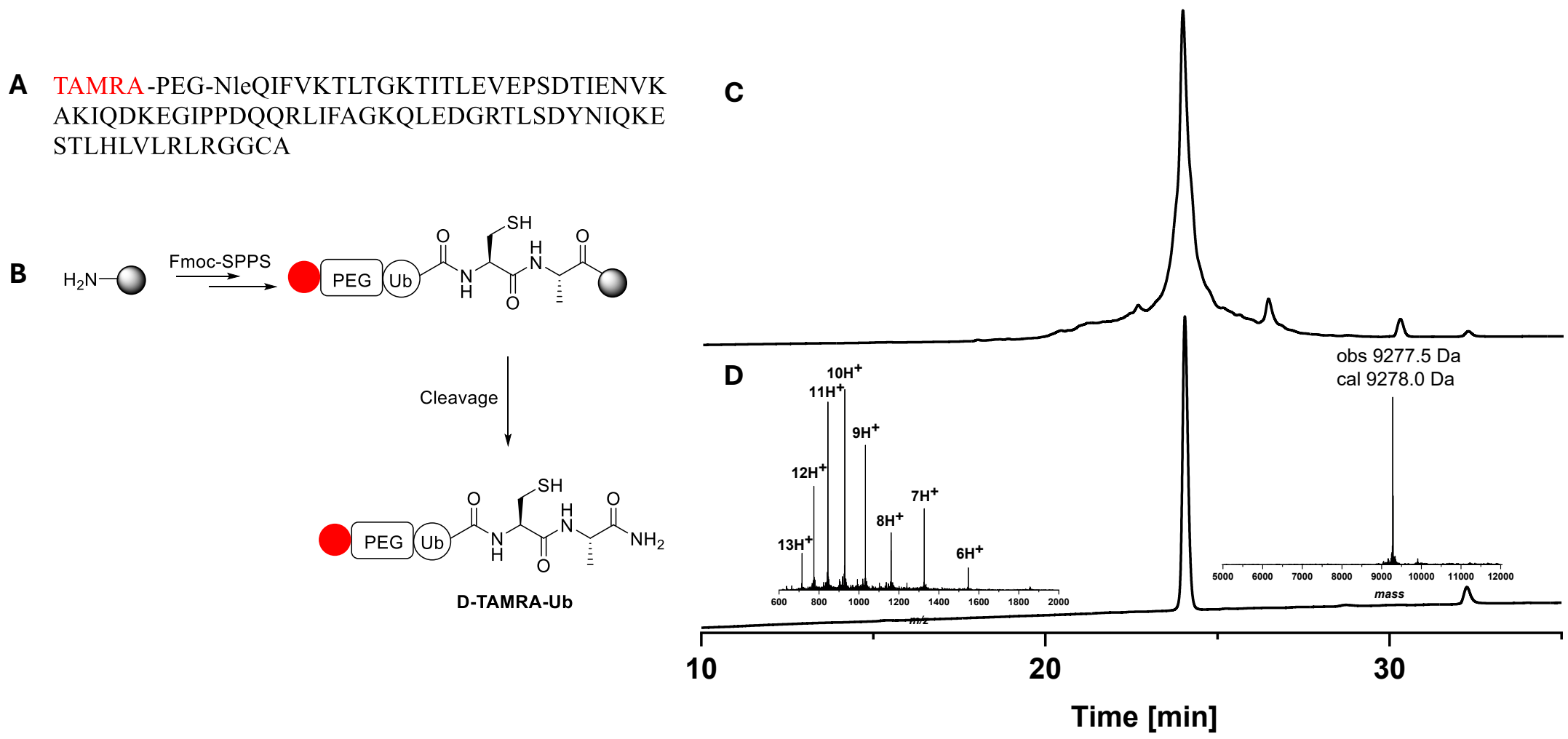

Figure S6. Synthesis of D-TAMRA-Ub (5) **A**) Sequence of D-TAMRA-Ub. (5) **B**) Schematic representation of synthetic route for D-TAMRA-Ub. **C**) HPLC of crude D-TAMRA-Ub. **D)** HPLC

and mass analysis of purified D-TAMRA-Ub with the observed mass 9277.5 ± 0.9 Da (calcd. 9278.0 Da, average isotopes).

D-TAMRA-Ub was synthesized on rink amide resin (0.2 mmol) following the described general procedure of Fmoc-SPPS. The crude peptide was purified by preparative HPLC using a C18 column with a gradient of 5-65% B over 40 mins to afford the purified peptide in 22% yield.

9. Synthesis of cR10DABCYL CPP:

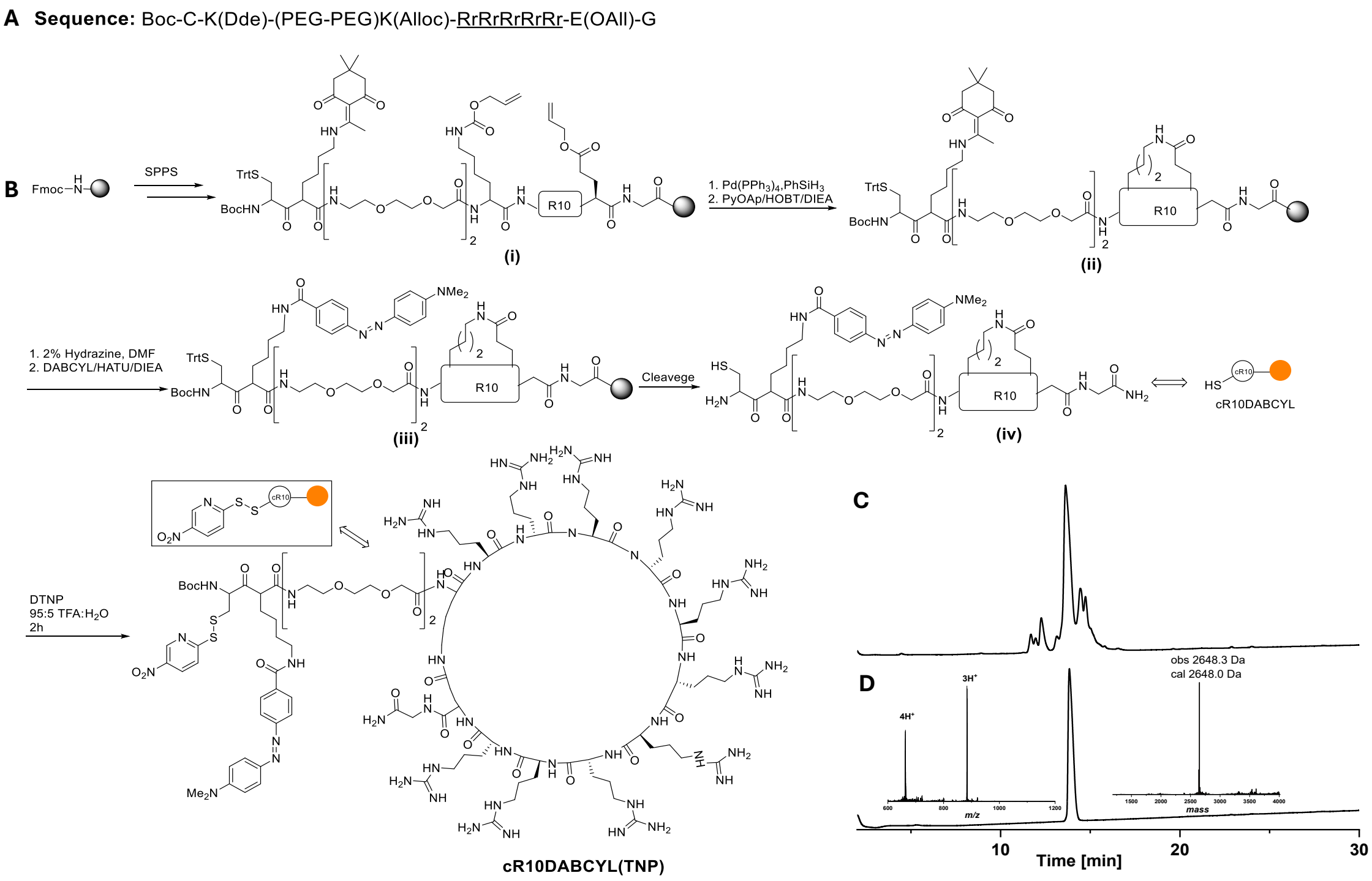

Figure S7. Synthesis of cell penetrating peptide cR10DABCYL. **A**) Sequence of cR10DABCYL, 'R'= L-arginine and 'r' = D-arginine. **B**) Schematic representation of synthetic route for cR10DABCYL. **C**) HPLC of crude cR10DABCYL. **C)** HPLC and mass analysis of purified cR10DABCYL with the observed mass 2648.3 ± 0.2 Da (calcd. 2648.0 Da, average isotopes).

cR10D was synthesized as previously reported by our group. Amino acids were assembled on pre-swollen Knorr resin (0.2 mmol g^{-1}) using an automated peptide synthesizer under standard Fmoc solid-phase peptide synthesis (SPPS) conditions to afford intermediate i; all arginine residues were double-coupled. Alloc and OAllyl protecting groups were simultaneously removed using $Pd(PPh_3)_4$ under the reported conditions, followed by on-

resin cyclization using PyAOP (5 equiv.), HOBt (5 equiv.), and DIEA (10 equiv.) in DMF for 1 h to yield intermediate ii. Subsequently, the Dde protecting group was removed by three treatment cycles with 2% hydrazine in DMF (30 min per cycle), after which DABCYL was coupled using DABCYL (4 equiv.), HATU (4 equiv.), and DIEA (8 equiv.) for 2 h to afford intermediate iii. The peptide was then cleaved from the resin as described above and purified by preparative HPLC on a C18 column using a linear gradient of 0–65% solvent B over 40 min to give the corresponding cR10D peptide iv. Activation of the cysteine residue in iv was achieved by treatment with DTNP (20 equiv.) in 95% TFA/H_2O for 2 h. The product was subsequently washed with DCM and cold diethyl ether, redissolved in 50% acetonitrile in water, and lyophilized to afford cR10D(TNP) in approximately 19% overall yield. No further purification was required for this activated CPP.

10. Conjugation reactions between cargos and CPP:

To conjugate cR10DABCYL(TNP) (Cys functionalized) with model cargos, both the peptide and cR10DABCYL(TNP) were dissolved in 6M Gn·HCl (pH=7.2) to a final concentration of 2mM. The solution containing the CPP was then added to the peptide solution, and the mixture was maintained at 37 °C for 5 minutes. Subsequently, the reaction mixture was purified utilizing a C18 semi-preparative column with a gradient of 5-65% B in 40 minutes with flow rate of 4 mL/min to yield the corresponding product.

10.1 Synthesis of L-TAMRA-ST-CPP:

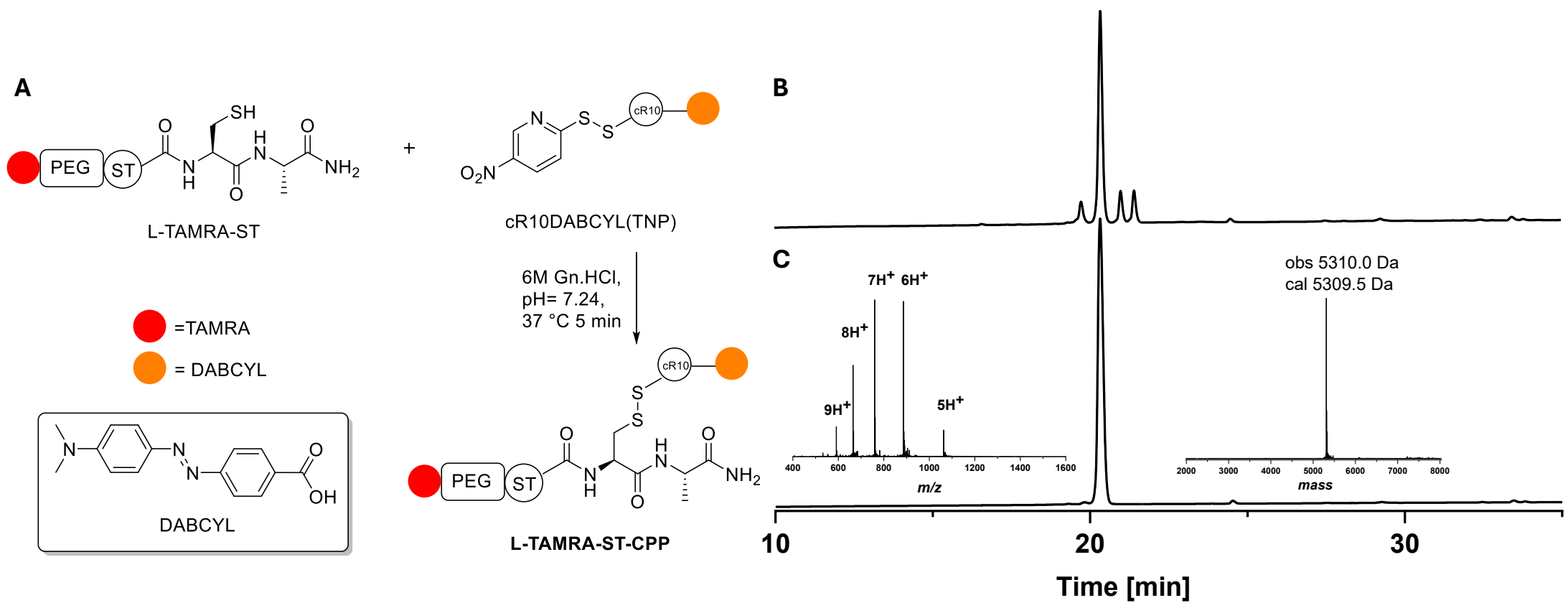

Figure S8. Conjugation of L-TAMRA-ST with cR10DABCYL(TNP). **A**) Schematic representation of synthetic route for L-TAMRA-ST-CPP. **B)** Conjugation reaction of L-TAMRA-ST with cR10DABCYL(TNP). **C)** HPLC and mass analysis of purified L-TAMRA-ST-CPP with the observed mass 5310.0 ± 0.6 Da (calc 5309.5 Da, average isotopes) and 61% yield.

10.2 Synthesis of D-TAMRA-ST-CPP:

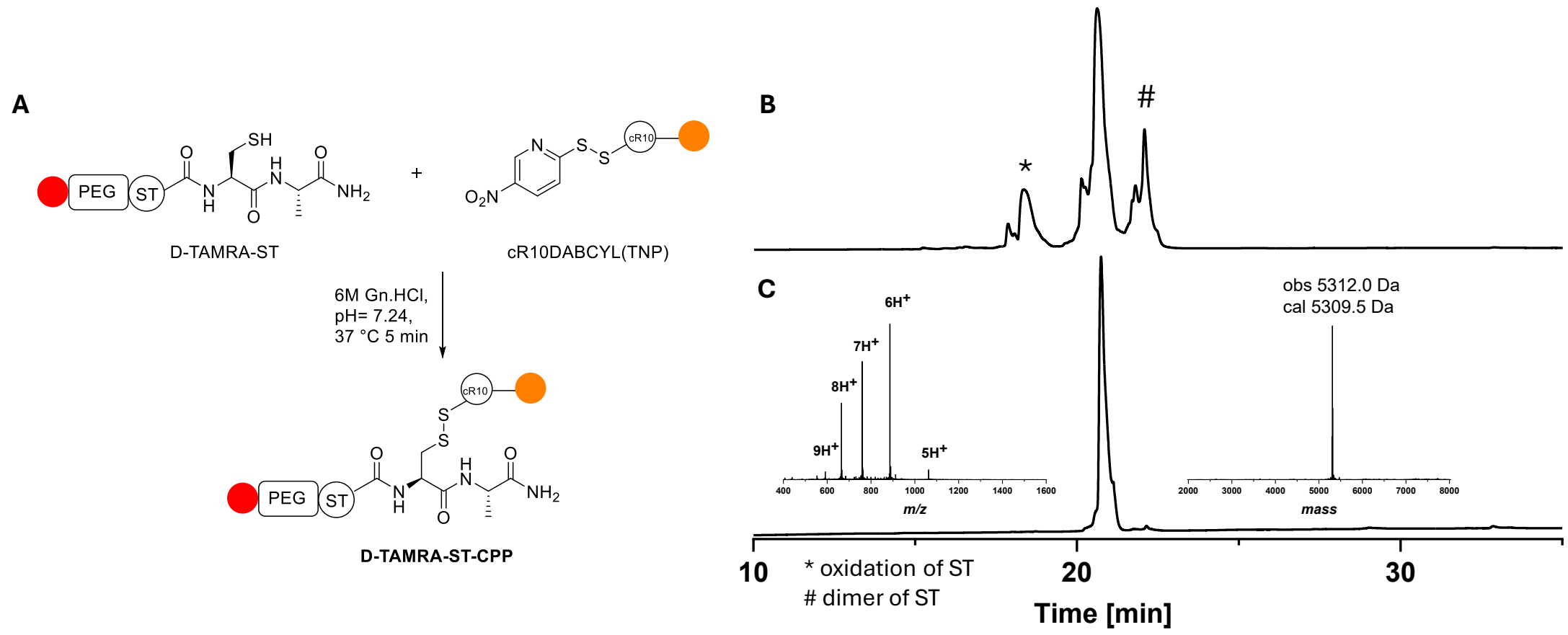

Figure S9. Conjugation of D-TAMRA-ST with cR10DABCYL(TNP). **A)** Schematic representation of synthetic route for D-TAMRA-ST-CPP. **B)** Conjugation reaction of D-TAMRA-ST with cR10DABCYL(TNP). **C)** HPLC and mass analysis of purified D-TAMRA-ST-CPP with the observed mass 5312.0 ± 0.7 Da (calc 5309.5 Da, average isotopes) and 43% yield.

10.3 Synthesis of L-TAMRA-FW1-ST-CPP:

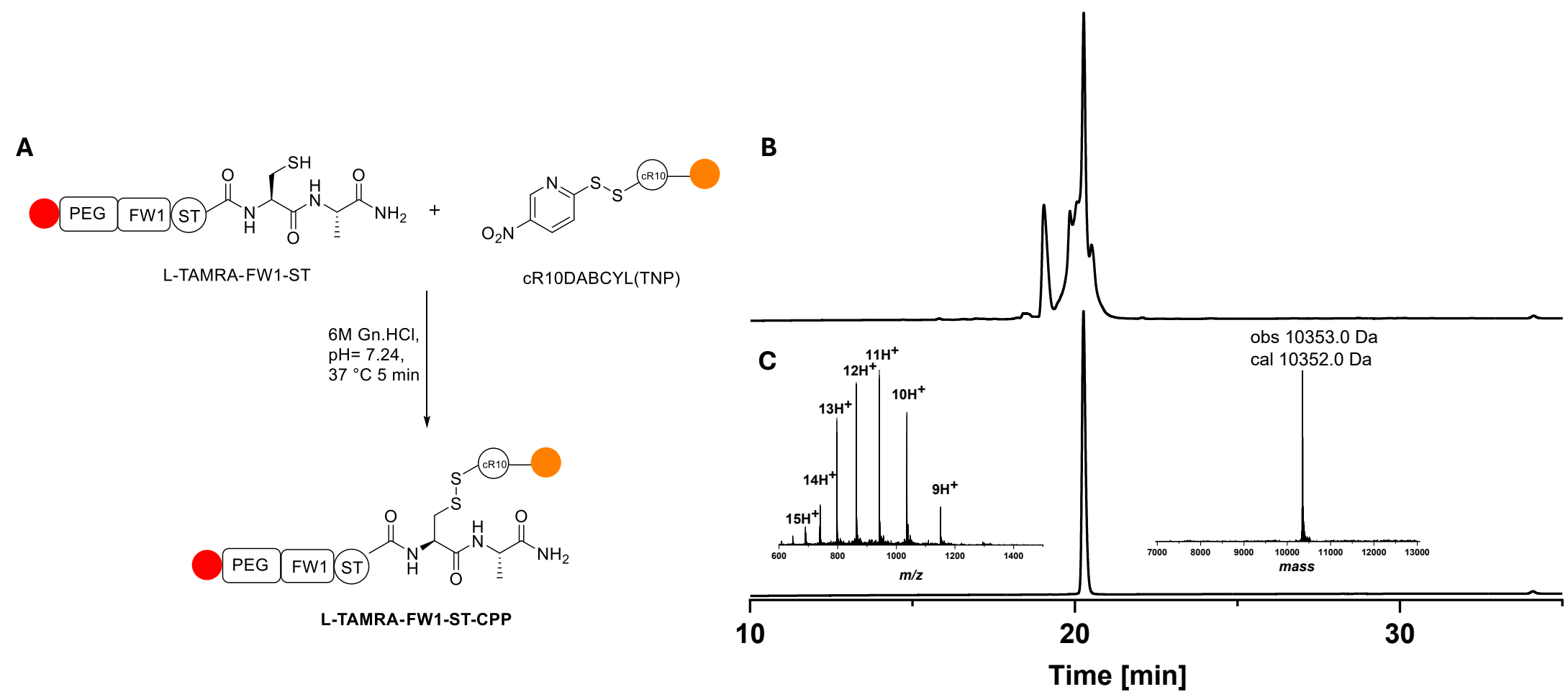

Figure S10. Conjugation of L-TAMRA-FW1-ST with cR10DABCYL(TNP). **A)** Schematic representation of synthetic route for L-TAMRA-FW1-ST-CPP. **B)** Conjugation reaction of L-TAMRA-FW1-ST with cR10DABCYL(TNP). **C)** HPLC and mass analysis of purified L-TAMRA-FW1-ST-CPP with the observed mass 10353.0 ± 1.0 Da (calc 10352 Da, average isotopes) and 47% yield.

10.4 Synthesis of D-TAMRA-FW1-ST-CPP:

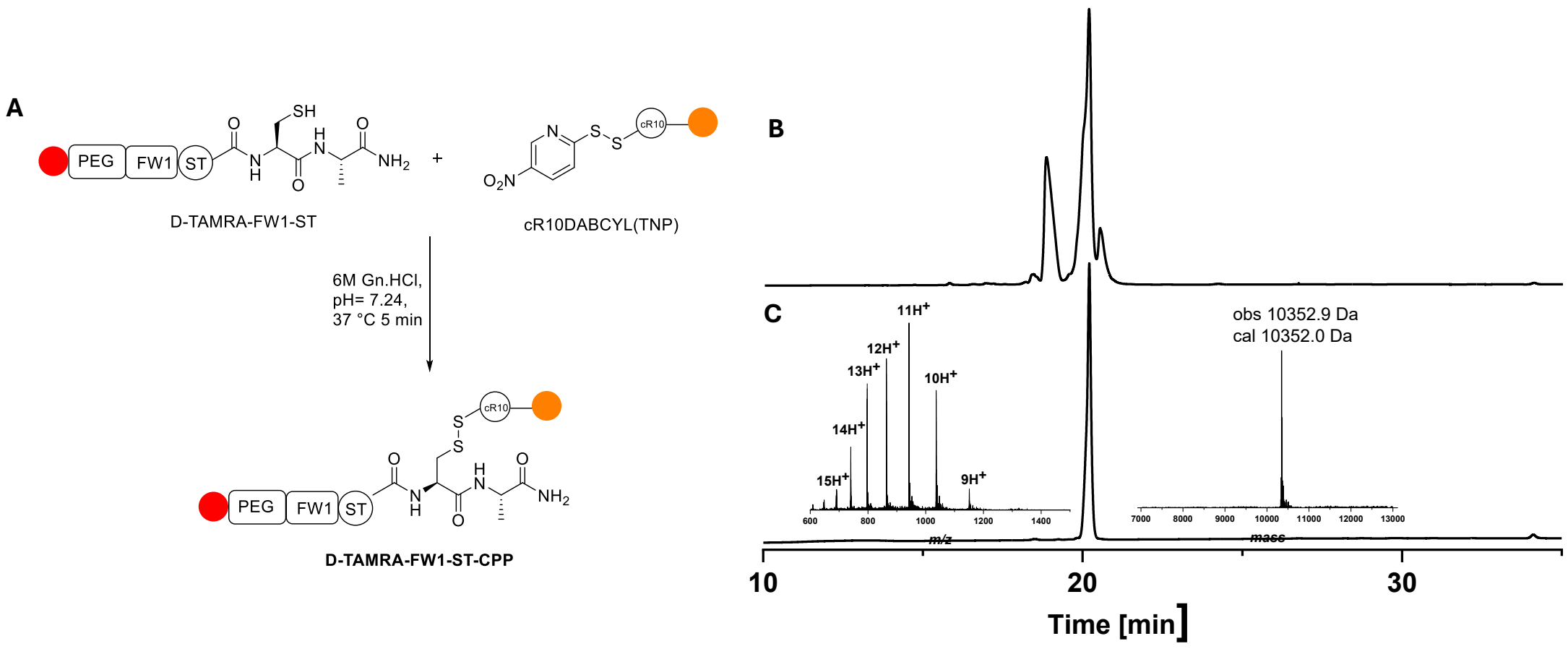

Figure S11. Conjugation of D-TAMRA-FW1-ST with cR10DABCYL(TNP). **A**) Schematic representation of synthetic route for D-TAMRA-FW1-ST-CPP. **B)** Conjugation reaction of D-TAMRA-FW1-ST with cR10DABCYL(TNP). **C)** HPLC and mass analysis of purified D-TAMRA-FW1-ST-CPP with the observed mass 10353.4 ± 1.0 Da (calc 10352 Da, average isotopes) and 39% yield.

10.5 Synthesis of L-TAMRA-Ub-CPP:

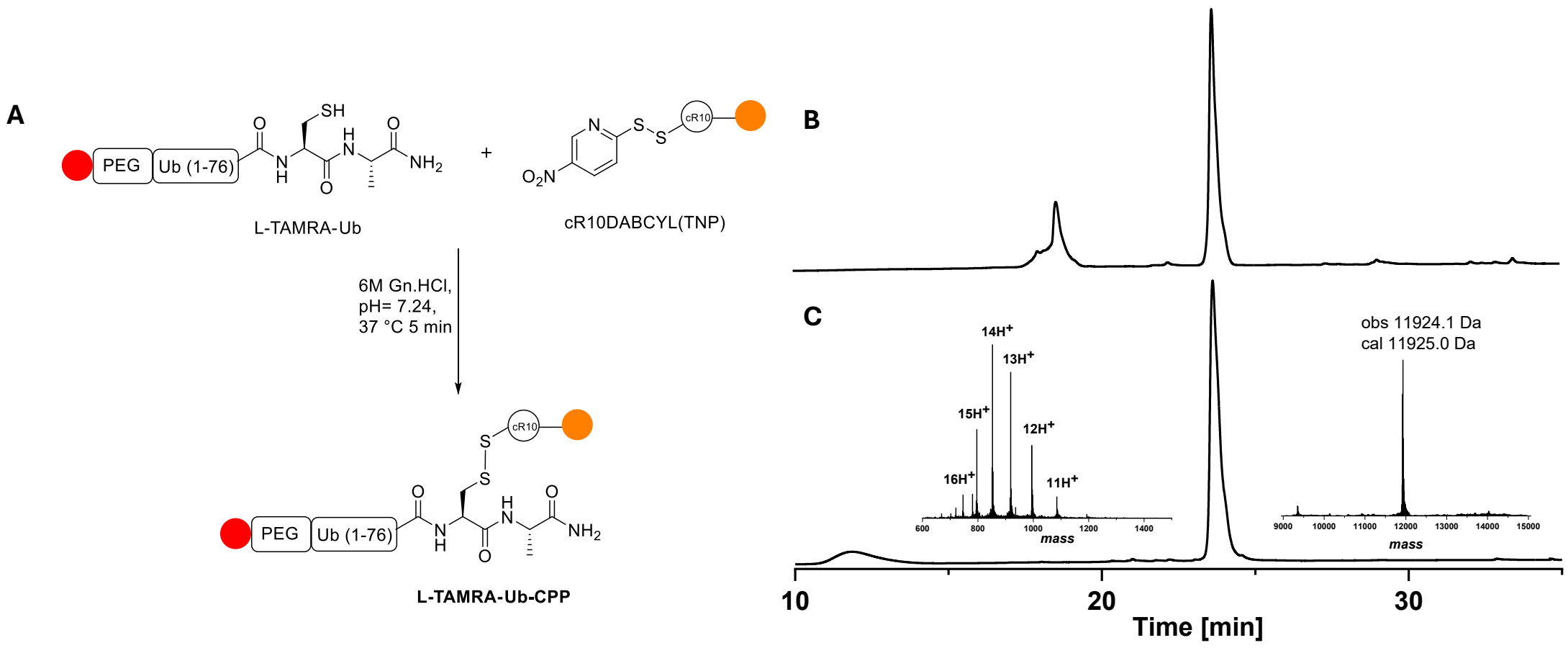

Figure S12. Conjugation of L-TAMRA-Ub with cR10DABCYL(TNP). **A)** Schematic representation of synthetic route for L-TAMRA-Ub-CPP. **B)** Conjugation reaction of L-TAMRA-Ub with cR10DABCYL(TNP). **C)** HPLC and mass analysis of purified L-TAMRA-Ub-CPP with the observed mass 11924.1 ± 1.0 Da (calc 10925.0 Da, average isotopes) and 65% yield.

10.6 Synthesis of D-TAMRA-Ub-CPP:

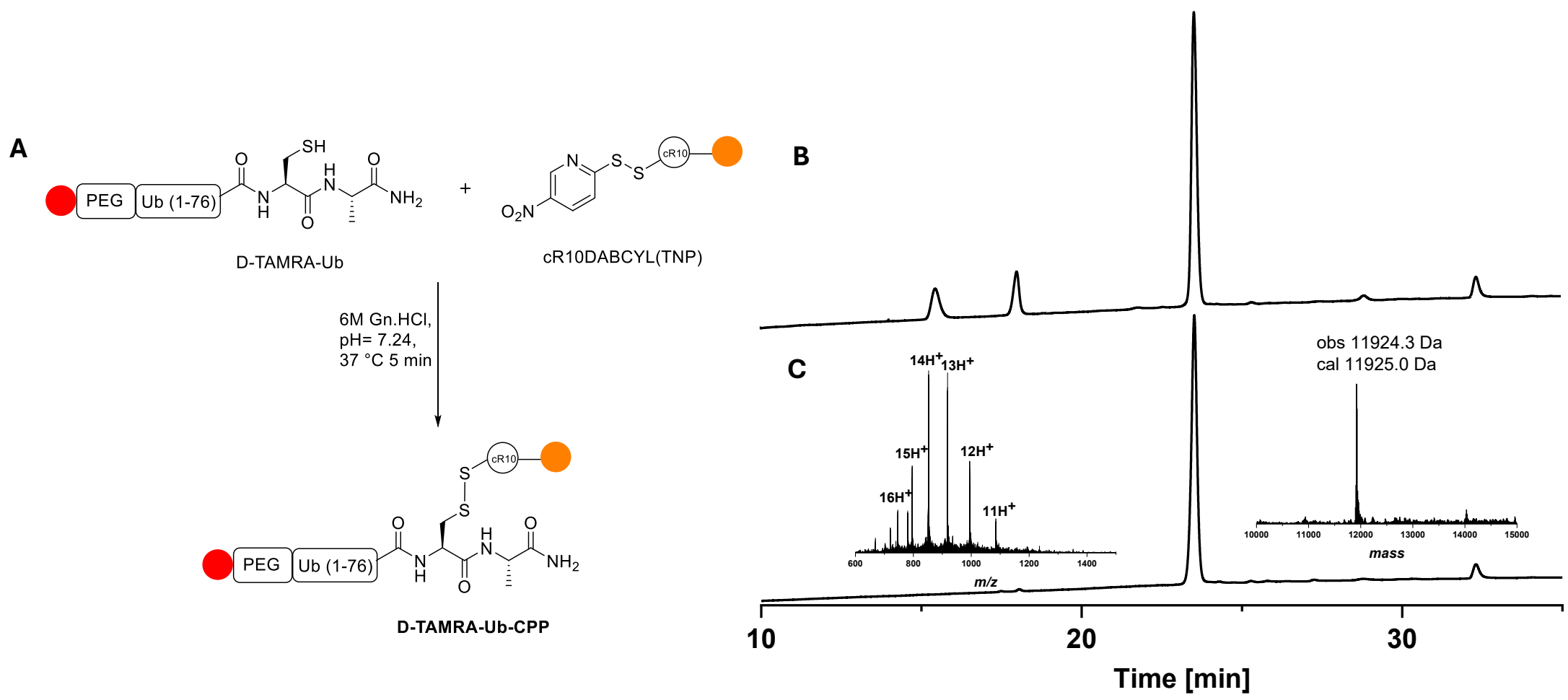

Figure S13. Conjugation of D-TAMRA-Ub with cR10DABCYL(TNP). **A)** Schematic representation of synthetic route for D-TAMRA-Ub-CPP. **B)** Conjugation reaction of D-TAMRA-Ub with cR10DABCYL(TNP). **C)** HPLC and mass analysis of purified D-TAMRA-Ub-CPP with the observed mass 11924.3 ± 1.4 Da (calc 11925.0 Da, average isotopes) and 61% yield.

11. Synthesis of Penetratin (Pen) CPP:

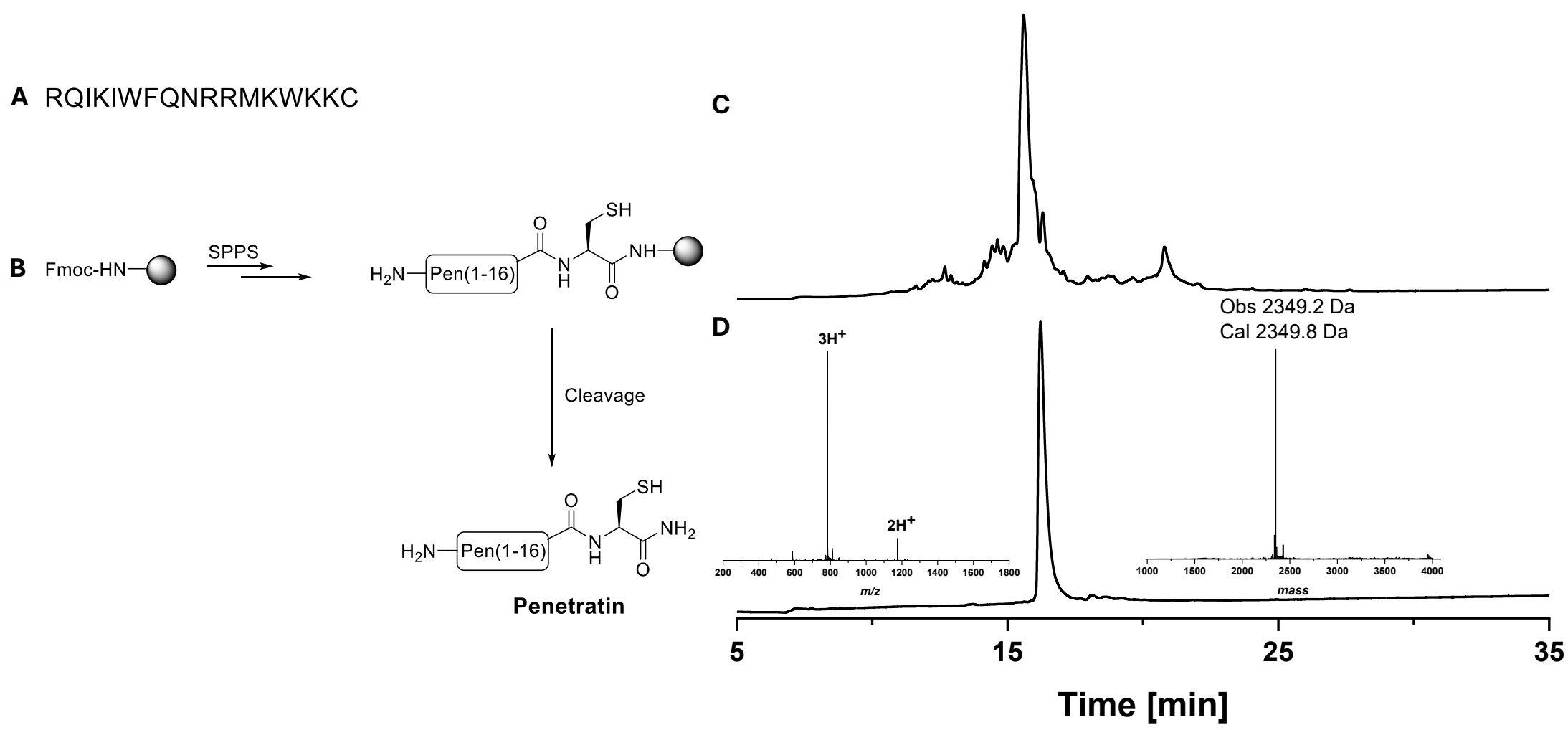

Figure S14. Synthesis of cell penetrating peptide Penetratin (Pen). **A**) Sequence of Pen. **B**) Schematic representation of synthetic route for Pen. **C**) HPLC of crude Pen. **D)** HPLC and mass analysis of purified Pen with the observed mass 2349.2 ± 0.5 Da (calc 2349.8 Da, average isotopes).

To synthesize Penetratin, amino acids were coupled on a pre-swollen Knorr Resin (0.2 mmol/g) utilizing an automated peptide synthesizer using the standard procedure. The peptide was then cleaved as mentioned above and purified by preparative HPLC using C18

column with a gradient of 0-65% B over 40 min to afford the corresponding Pen in ~22 % yield**.** Activation of the cysteine residue was achieved by treatment with DTNP (1 equiv.) in 95% TFA/H_2O for 2 h. The product was subsequently washed with DCM and cold diethyl ether, redissolved in 50% acetonitrile in water, and lyophilized. Following, the activated Pen was purified by preparative using a C18 column with a gradient of 0-65% B over 40 min to afford the corresponding Pen(TNP) in ~80 % yield.

12. Conjugation reactions between cargos and Pen CPP:

To conjugate Pen(TNP) (Cys functionalized) with model cargos, both the peptide and Pen(TNP) were dissolved in 6M Gn·HCl (pH=7.2) to a final concentration of 2mM. The solution containing the CPP was then added to the peptide solution, and the mixture was maintained at 37 °C for 5 minutes. Subsequently, the reaction mixture was purified utilizing a C18 semi-preparative column with a gradient of 5-65% B in 40 minutes with flow rate of 4 mL/min to yield the corresponding product.

12.1 Synthesis of L-TAMRA-ST-Pen:

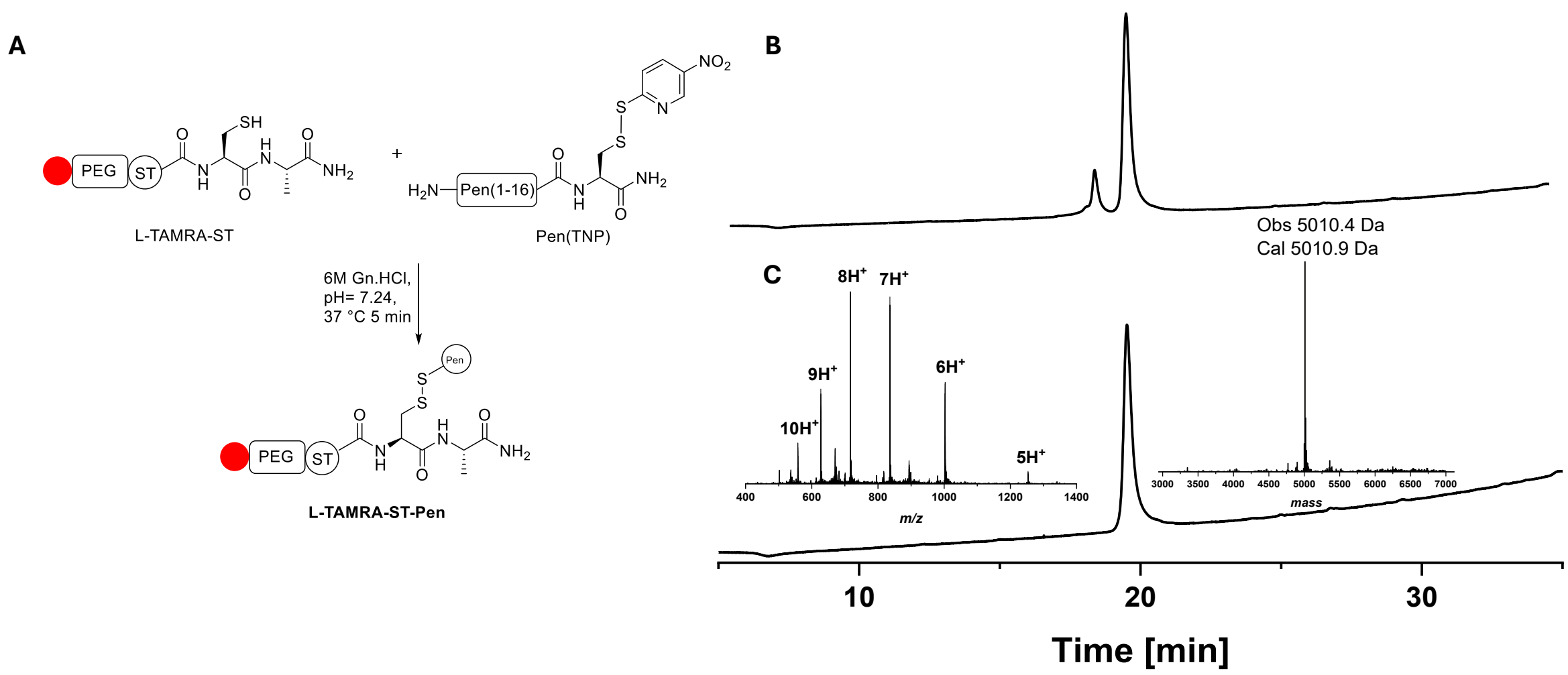

Figure S15. Conjugation of L-TAMRA-ST with Pen(TNP). **A**) Schematic representation of synthetic route for L-TAMRA-ST-Pen. **B)** Conjugation reaction of L-TAMRA-ST with Pen(TNP). **C)** HPLC and mass analysis of purified L-TAMRA-ST-Pen with the observed mass 5010.4 ± 0.5 Da (calc 5010.9 Da, average isotopes) and 72% yield.

12.2 Synthesis of D-TAMRA-ST-Pen:

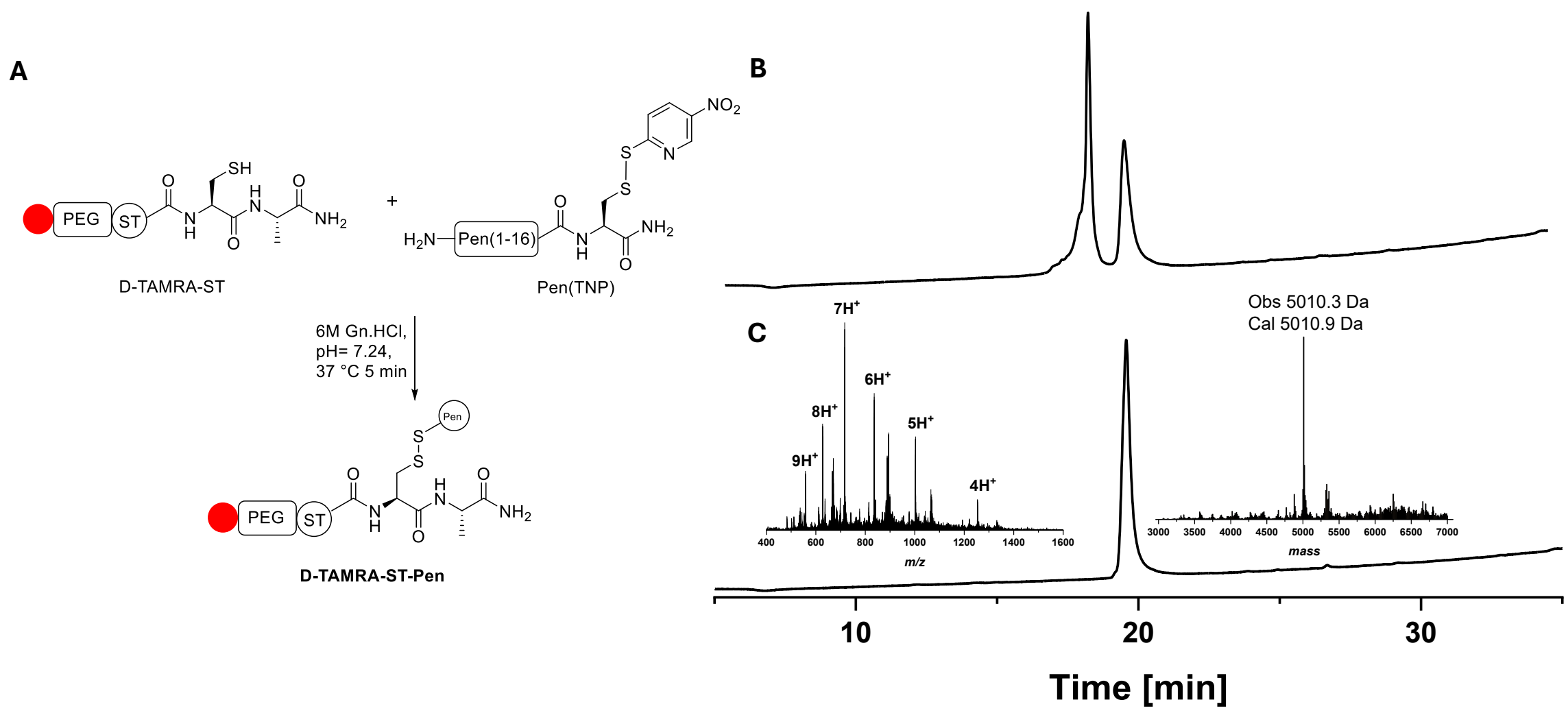

Figure S16. Conjugation of D-TAMRA-ST with Pen(TNP). **A**) Schematic representation of synthetic route for D-TAMRA-ST-Pen. **B)** Conjugation reaction of D-TAMRA-ST with Pen(TNP). **C)** HPLC and mass analysis of purified L-TAMRA-ST-Pen with the observed mass 5010.3 ± 0.5 Da (calc 5010.9 Da, average isotopes) and 73% yield.

12.3 Synthesis of L-TAMRA-FW1-ST-Pen:

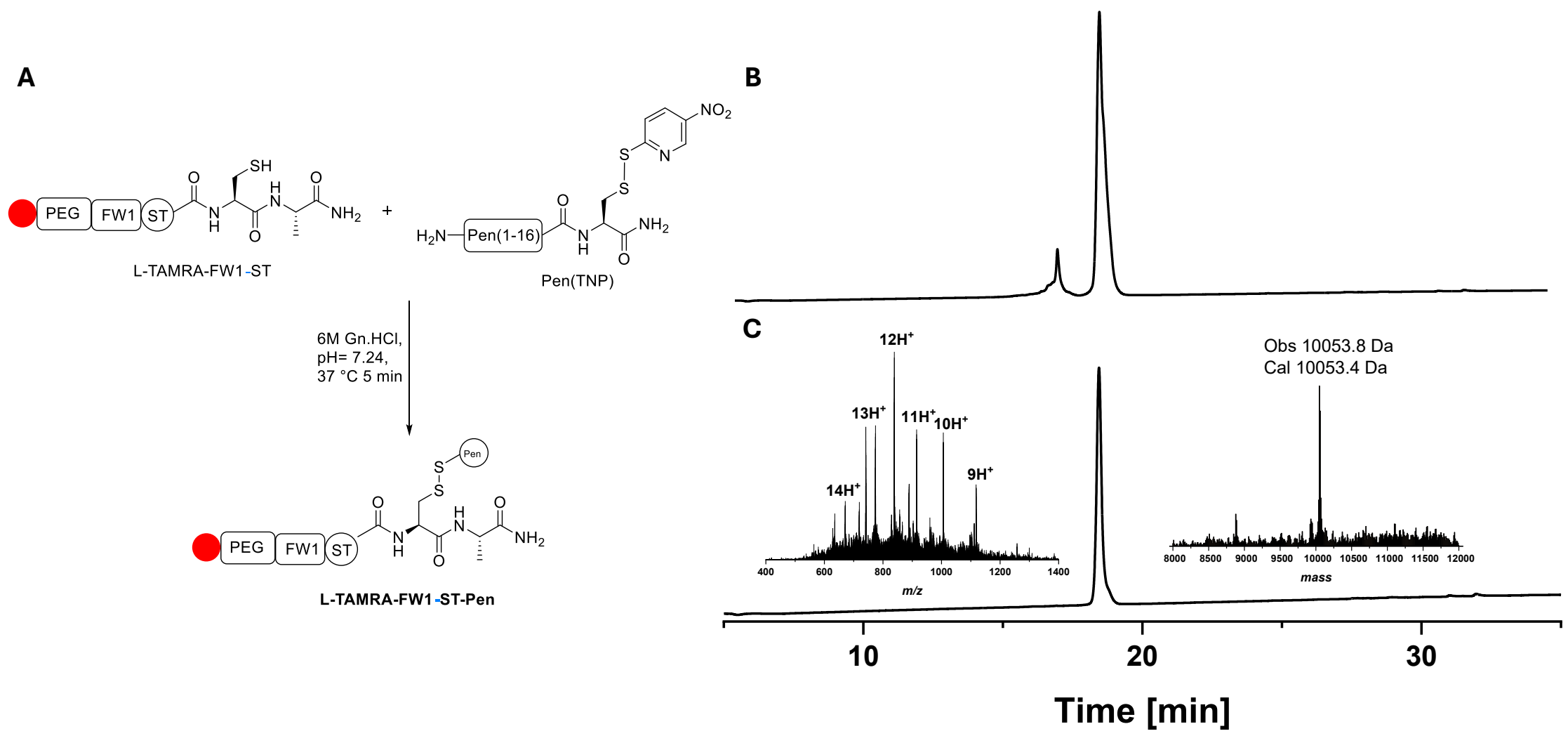

Figure S17. Conjugation of L-TAMRA-FW1-ST with Pen(TNP). **A**) Schematic representation of synthetic route for L-TAMRA-FW1-ST-Pen. **B)** Conjugation reaction of L-TAMRA-FW1-ST with Pen(TNP). **C)** HPLC and mass analysis of purified L-TAMRA-FW1-ST-Pen with the observed mass 10053.8 ± 0.5 Da (calc 10053.4 Da, average isotopes) and 67% yield.

12.4 Synthesis of D-TAMRA-FW1-ST-Pen:

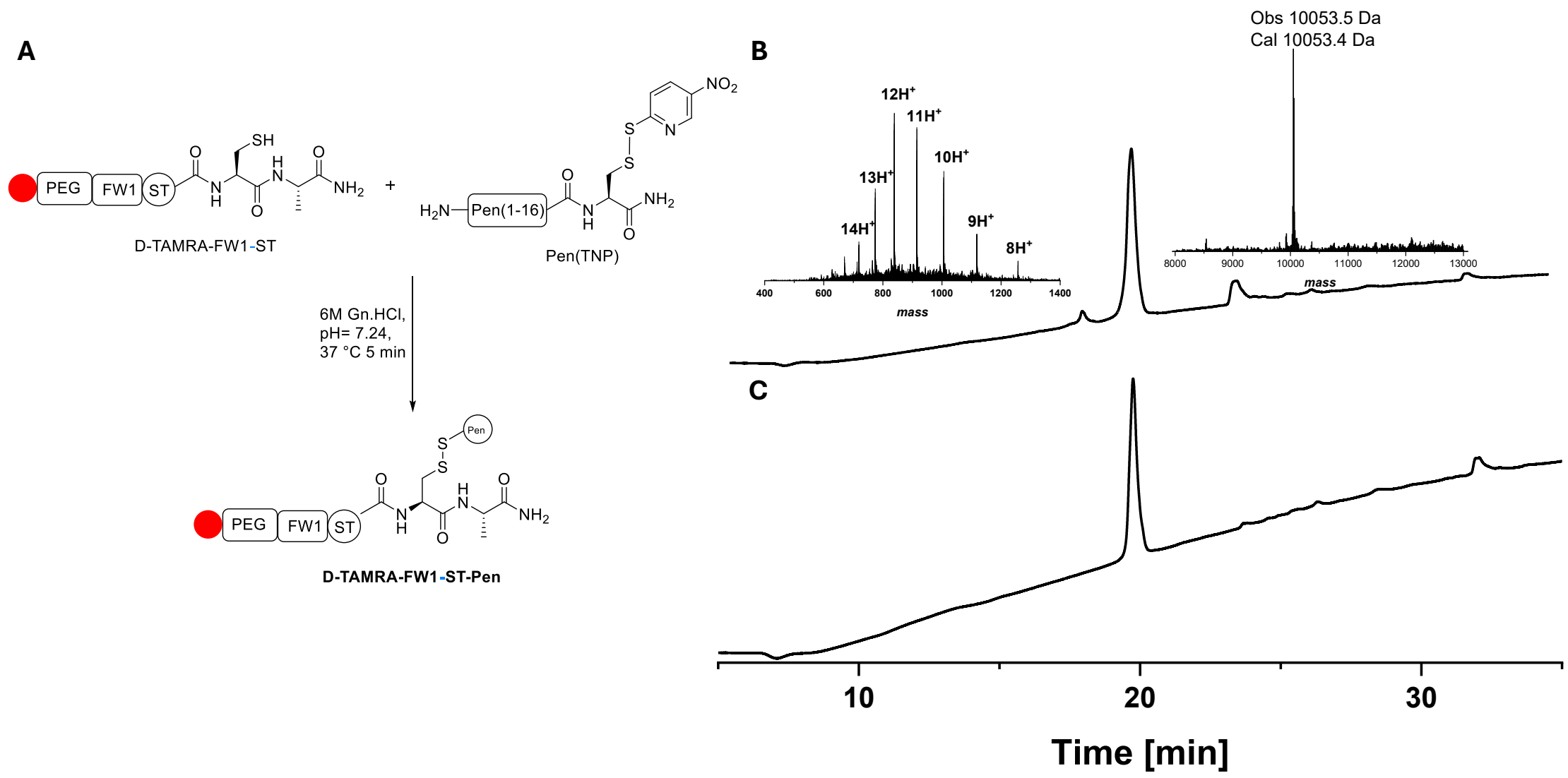

Figure S18. Conjugation of D-TAMRA-FW1-ST with Pen(TNP). **A**) Schematic representation of synthetic route for D-TAMRA-FW1-ST-Pen. **B)** Conjugation reaction of L-TAMRA-FW1-ST with Pen(TNP). **C)** HPLC and mass analysis of purified D-TAMRA-FW1-ST-Pen with the observed mass 10053.5 ± 0.5 Da (calc 10053.4 Da, average isotopes) and 63% yield.

Synthesis of L-TAMRA-Ub-Pen:

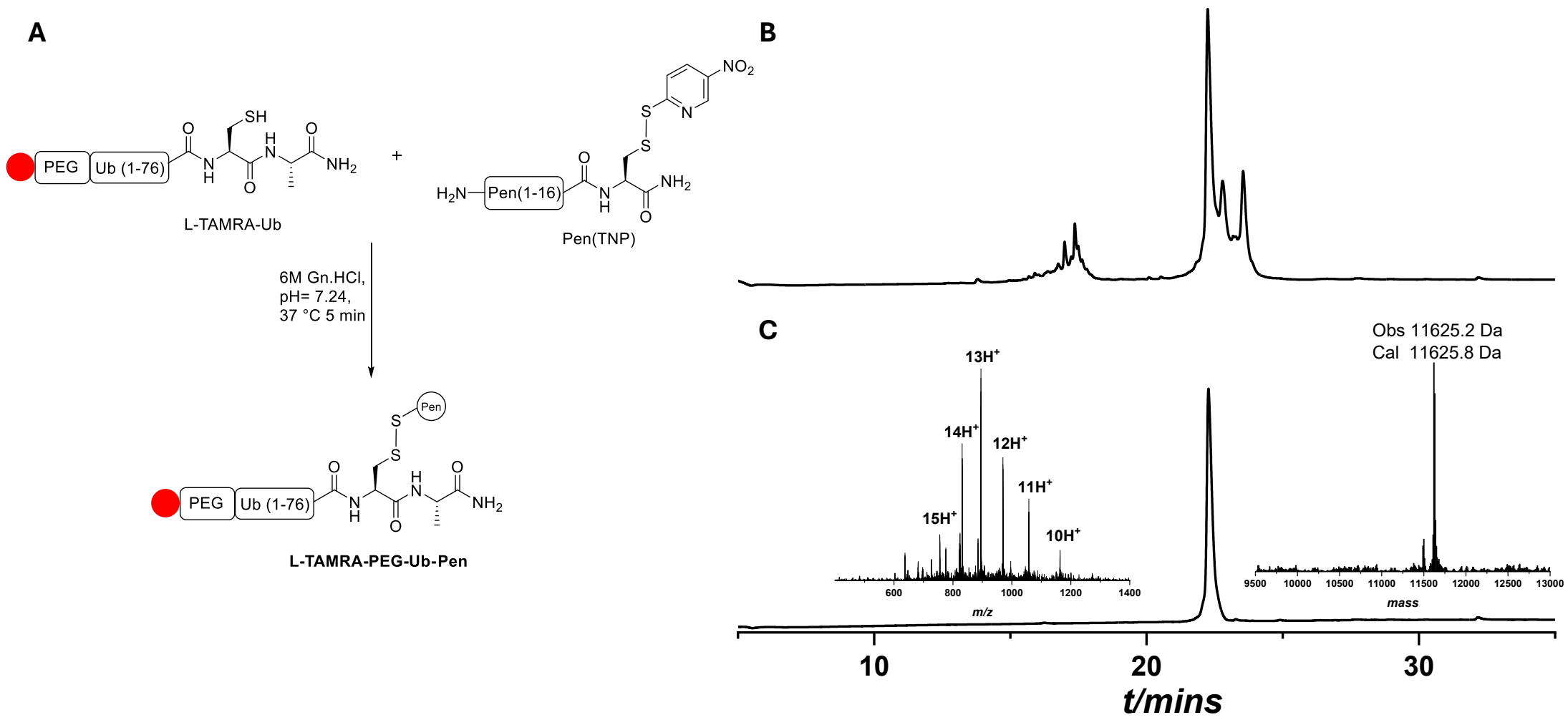

Figure S19. Conjugation of L-TAMRA-Ub with Pen(TNP). **A**) Schematic representation of synthetic route for L-TAMRA-Ub-Pen. **B)** Conjugation reaction of L-TAMRA-Ub with Pen(TNP). **C)** HPLC and mass analysis of purified L-TAMRA-Ub-Pen with the observed mass 11625.2 ± 0.5 Da (calc 11625.8 Da, average isotopes) and 59% yield.

12.5 Synthesis of D-TAMRA-Ub-Pen:

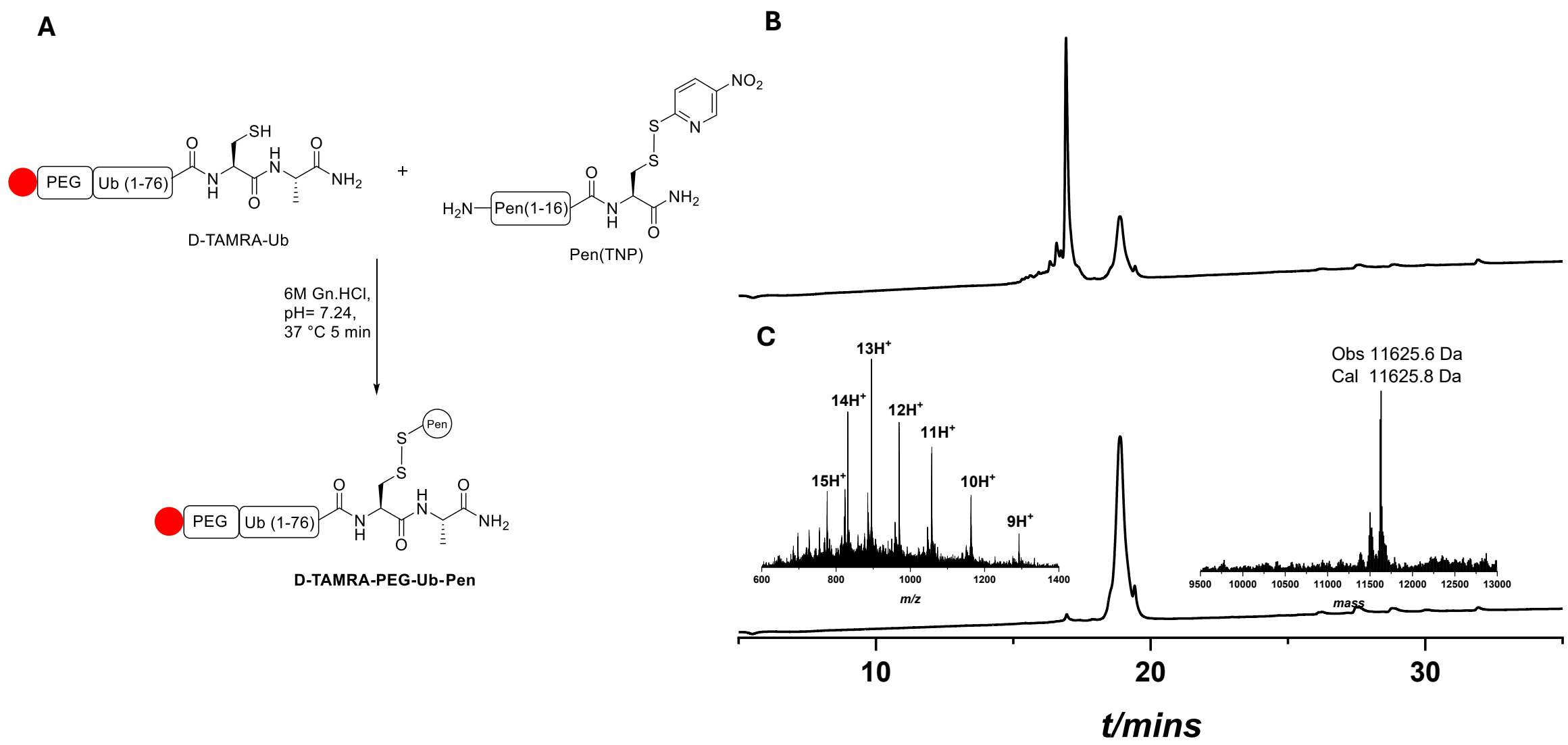

Figure S20. Conjugation of D-TAMRA-Ub with Pen(TNP). **A)** Schematic representation of synthetic route for D-TAMRA-Ub-Pen. **B)** Conjugation reaction of D-TAMRA-Ub with Pen(TNP). **C)** HPLC and mass analysis of purified D-TAMRA-Ub-Pen with the observed mass 11625.6 ± 0.5 Da (calc 11625.8 Da, average isotopes) and 62% yield.

13. Circular dichroism (CD) measurement:

Circular dichroism (CD) measurements were performed at 25 °C in 10 mM sodium phosphate buffer, pH 7, containing ~5% acetonitrile for protein solubility. Samples were measured in a 0.1 cm path-length cuvette using a Jasco J-810 spectropolarimeter and Applied Photophysics Chirascan, over a wavelength range of 180–300 nm. Spectra were recorded by averaging five scans with 1 nm intervals. The ellipticity (θ, in millidegrees) was converted to mean residue molar ellipticity ([θ], deg·cm^2·dmol^{-1}) using the following equation:

$$[\theta]_{MER} = \frac{\theta\ (mdeg)}{10\ x\ l\ (cm)\ x\ N\ (\#amide\ bonds) x\ C\ (M)}$$

14. General procedure for cellular uptake studies using flow cytometry:

Proteins were dissolved in DMSO, and their concentrations were determined using both a NanoDrop spectrophotometer and a Bradford assay against a BSA calibration curve. Each DMSO stock was then adjusted to the same concentration for the L- and D-cargo variants, and HPLC was used to confirm comparable peak heights between the enantiomeric samples. For

flow cytometry analysis, cells were seeded as described above. Prior to treatment, the growth medium was aspirated, and HeLa and U2OS cells were washed once with sterile calcium- and magnesium-containing PBS. Protein solutions were diluted in complete culture medium to the desired final concentrations, incubated at 37 °C to allow protein folding, and added to the cells. Cells were incubated for 1 h at 37 °C in a humidified incubator with 5% CO_2. Following incubation, the medium was aspirated, and cells were washed with calcium- and magnesium-free PBS, detached, and collected by centrifugation at 500 × g for 5 min. Cells were washed three additional times with calcium- and magnesium-free PBS to remove extracellular or surface-bound material, and the final cell pellet was resuspended in 200 µL calcium- and magnesium-free PBS for flow cytometry analysis.

Cellular uptake of protein cargos was analyzed using a Cytek Aurora spectral flow cytometer (Cytek Biosciences) equipped with four excitation lasers (405, 488, 561, and 640 nm) and full-spectrum detection across all emission channels. TAMRA fluorescence was excited using the 561 nm laser, and emission spectra were collected across the appropriate detector range. Samples were acquired at a flow rate of 15–20 µL min^{-1}, and at least 5000 single-cell events were recorded per sample. Forward scatter (FSC) versus side scatter (SSC) plots were used to gate the main cell population and exclude cellular debris. Doublets were excluded using FSC-A versus FSC-H plots. Final quantification was performed on the TAMRA-positive single-cell population.

For flow cytometry analysis of Jurkat cells, suspension cultures were collected directly without detachment. Cells were pelleted by centrifugation at 200 × g for 5 min, washed once with calcium- and magnesium-containing PBS, and resuspended in complete RPMI-1640 medium. Protein solutions were diluted in complete medium to the desired final concentrations and added to the cells, followed by incubation for 1 h at 37 °C in a humidified incubator with 5% CO_2. After incubation, cells were collected by centrifugation at 200 × g for 5 min and washed three times with calcium- and magnesium-free PBS to remove extracellular or surface-associated material. The final cell pellet was resuspended in 200 µL calcium- and magnesium-free PBS and analyzed by spectral flow cytometry as described above.

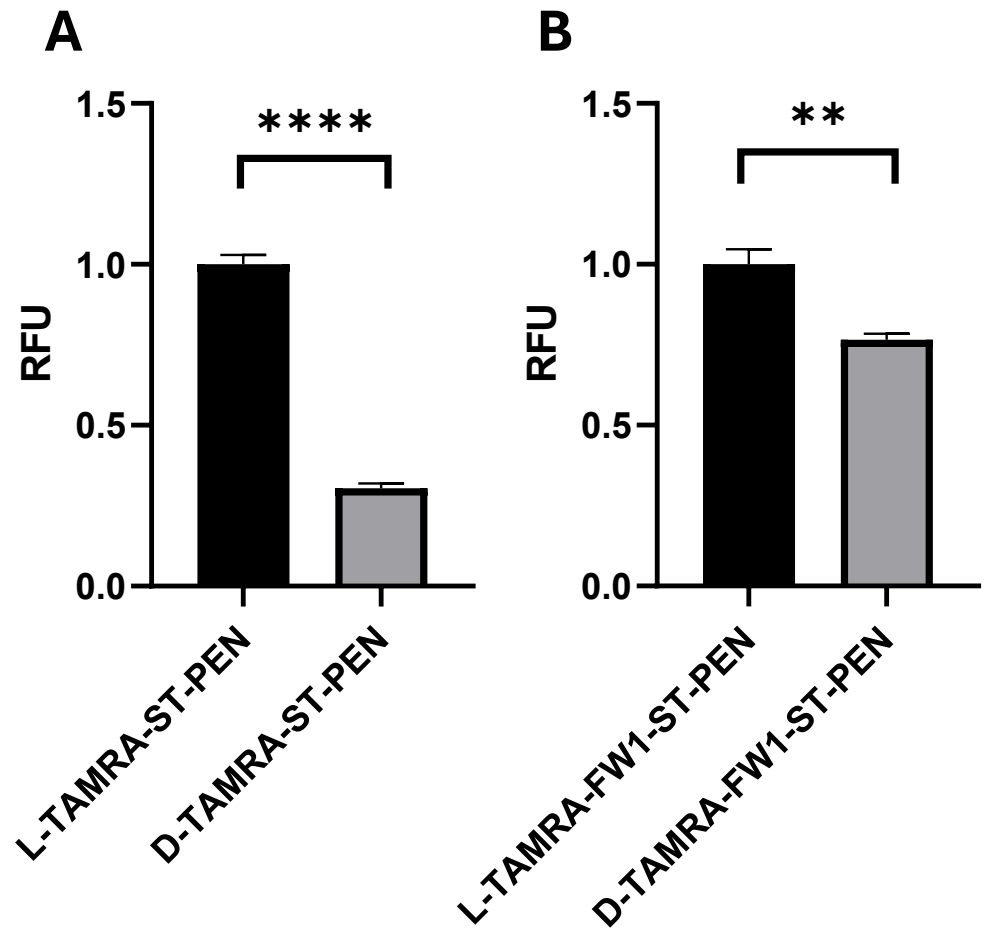

Figure S21. Flow cytometry analysis of TAMRA fluorescence intensities reporting the cellular uptake of each cargo and its mirror-image analogue linked to Pen CPP in U2OS cells at a concentration of 2 μM following 1 hour of incubation. A) Quantification of uptake of **L-** and **D-TAMRA-ST-Pen** and, B) quantification of uptake of **L-** and **D-TAMRA-FW1-ST-Pen**. Significance is indicated as: ns = not significant ($p \geq 0.05$), * $p < 0.05$, ** $p < 0.01$, *** $p < 0.001$, **** $p < 0.0001$.

15. General method for cell imaging using laser scanning and spinning-disk confocal microscopy and image analysis:

For confocal microscopy analysis, HeLa and U2OS cells were seeded on poly-L-lysine–coated Ibidi 8-well chamber slides at a density of 5×10^4 cells per well and cultured for 24 h to reach approximately 90% confluency. Prior to treatment, the growth medium was aspirated, and cells were washed three times with sterile calcium- and magnesium-containing PBS. Protein solutions were diluted in complete culture medium to the desired final concentrations and added to the cells, followed by incubation for 1 h at 37 °C in a humidified incubator with 5% CO_2. After incubation, cells were washed three times with calcium- and magnesium-containing PBS to remove extracellular or surface-associated material. For nuclear staining, cells were incubated with Hoechst 33342 (10 μM final concentration) in phenol-red–free Opti-DMEM supplemented with 10% FBS, 0.2 mM L-glutamine, and penicillin–streptomycin for 10 min. Cellular delivery and intracellular localization of fluorescent protein cargos were investigated using laser-scanning confocal microscopy (LSCM) on a Zeiss LSM 710 confocal microscope (Axio Observer) equipped with a Plan-Apochromat 63×/NA 1.4 oil-immersion objective for live-cell imaging. Imaging was performed at room temperature. Hoechst fluorescence was

excited using a 405 nm laser, and TAMRA fluorescence was excited using a 543 nm laser (10 mW). All channels were acquired with a 1 Airy unit pinhole setting, and acquisition parameters were kept constant across all samples. Representative images were analyzed using ZEN 3.0 (ZEN Lite, Zeiss) software. Fluorescence intensities were normalized using the range indicator tool based on a representative image, and identical intensity adjustments were uniformly applied to all images across different treatments. Importantly, L- and D-cargo images were processed using the same intensity scaling and analysis parameters, with no differential post-processing between enantiomeric samples.

In addition, cellular delivery and localization of fluorescent protein cargos were analyzed using a spinning-disk confocal microscope. Cells were prepared and treated as described above, including incubation with protein solutions and Hoechst 33342 (10 μM) in phenol-red–free medium for nuclear staining. Live-cell imaging was performed at room temperature using the spinning-disk system, with appropriate excitation lasers for Hoechst (405 nm) and TAMRA (561 nm). Imaging parameters, including laser power, exposure time, and detector settings, were kept constant across all samples. Z-stacks spanning the full cell volume were acquired to ensure accurate 3D representation of fluorescence. Representative image stacks were analyzed using Imaris 10.0.1 software. For quantification, the sum of TAMRA fluorescence intensity across the entire z-stack was measured and divided by the number of cells (determined from Hoechst-stained nuclei) to calculate the average fluorescence per cell. Intensity scaling and analysis parameters were applied identically to all samples, and L- and D-cargo images were processed using the same workflow to ensure unbiased comparison between enantiomers.

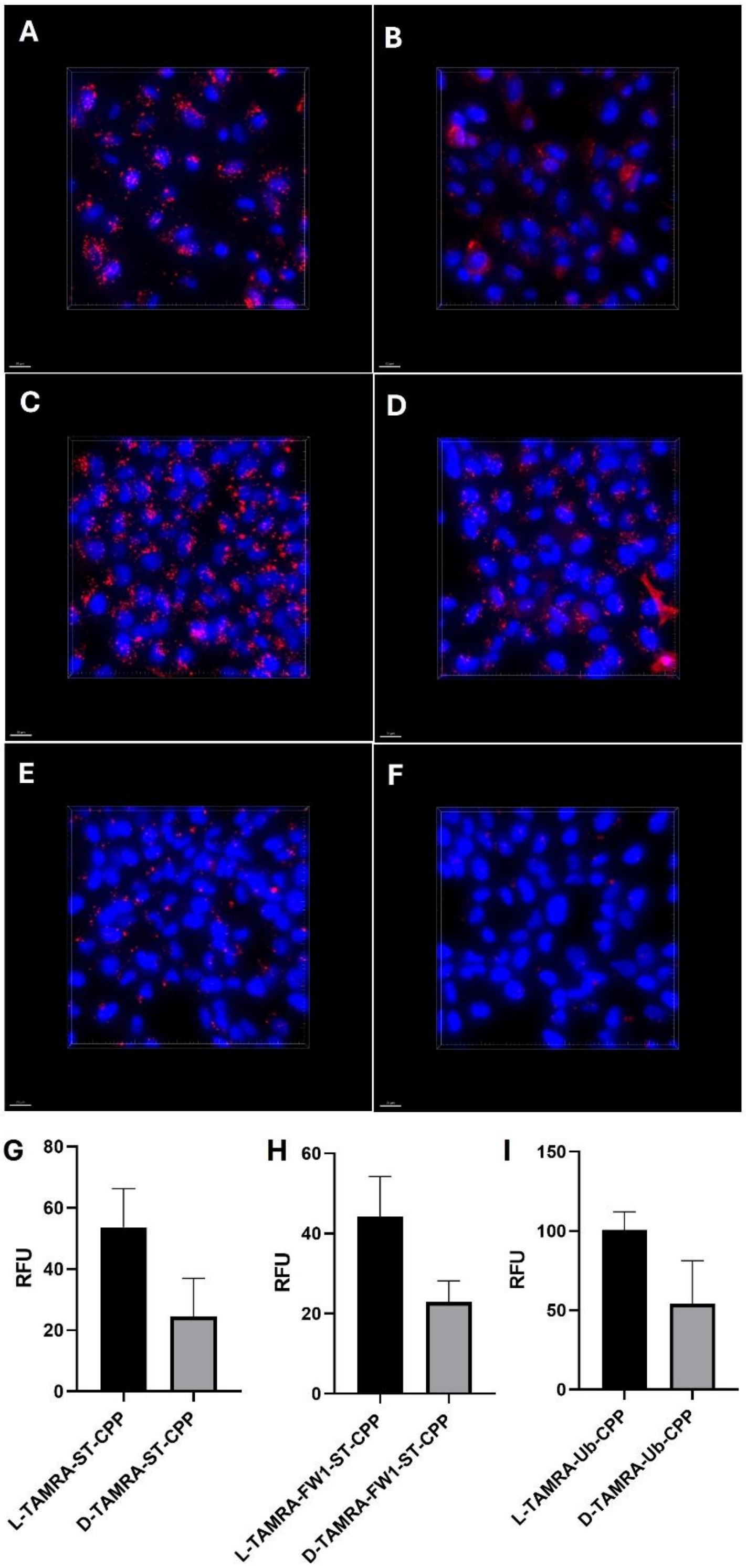

Figure S22. Representative z-stack projections of U2OS cells treated with: A) L-TAMRA-ST-CPP. B) D-TAMRA-ST-CPP. C) L-TAMRA-FW1-ST-CPP. D) D-TAMRA-FW1-ST-CPP. E) L-TAMRA-Ub-CPP. F) D-TAMRA-Ub-CPP. G) H) I) Cells were stained with Hoechst (blue) and imaged using a spinning-disk confocal microscope.

16. Lysis protocol using RIPA buffer:

Following protein delivery and three washes with calcium- and magnesium-free PBS, adherent HeLa and U2OS cells were incubated with 70 µL Radio-Immunoprecipitation Assay buffer (RIPA) buffer (50 mM Tris, pH 7.4; 150 mM NaCl; 0.5% deoxycholate; 0.1% SDS; 1% NP-40; 1 µL protease inhibitor cocktail) directly in the culture plate on ice for 20 min. The lysate was then collected into microcentrifuge tubes and kept on ice for an additional 20 min. Jurkat suspension cells were washed three times with cold PBS and resuspended in 70 µL RIPA buffer, followed by incubation on ice for 40 min. Lysates from both adherent and suspension cells were sonicated for 1 min, centrifuged at 13,000 × g for 10 min at 4 °C, and the supernatant collected. Protein concentrations were determined using a Bradford assay against a BSA calibration curve.

17. Fluorescence SDS-PAGE gel analysis and western blot:

Lysate samples were mixed with 6× reducing sample buffer and heated at 95 °C for 5 min prior to loading. 20 µg of protein per sample was loaded on 4–20% SDS-PAGE gels, which were imaged using an ECL detection system. Following electrophoresis, proteins were transferred onto PVDF membranes, which were then blocked with InstantBlock solution (Tivan-Biotech) for 10 min at room temperature. Membranes were incubated overnight at 4 °C with rabbit monoclonal anti-GAPDH (1:1000, Abcam, ab181602) as a loading control to ensure equal protein loading and accurate comparison between samples, followed by 1 h incubation with the corresponding secondary antibody at room temperature. After washing with TBST, membranes were treated with Immobilon Crescendo Western HRP substrate (Merck Millipore), and chemiluminescent signals were captured using the ECL detection system. Chemiluminescent signals were quantified using ImageJ (Fiji), with band intensities normalized to GAPDH to correct for differences in protein loading.

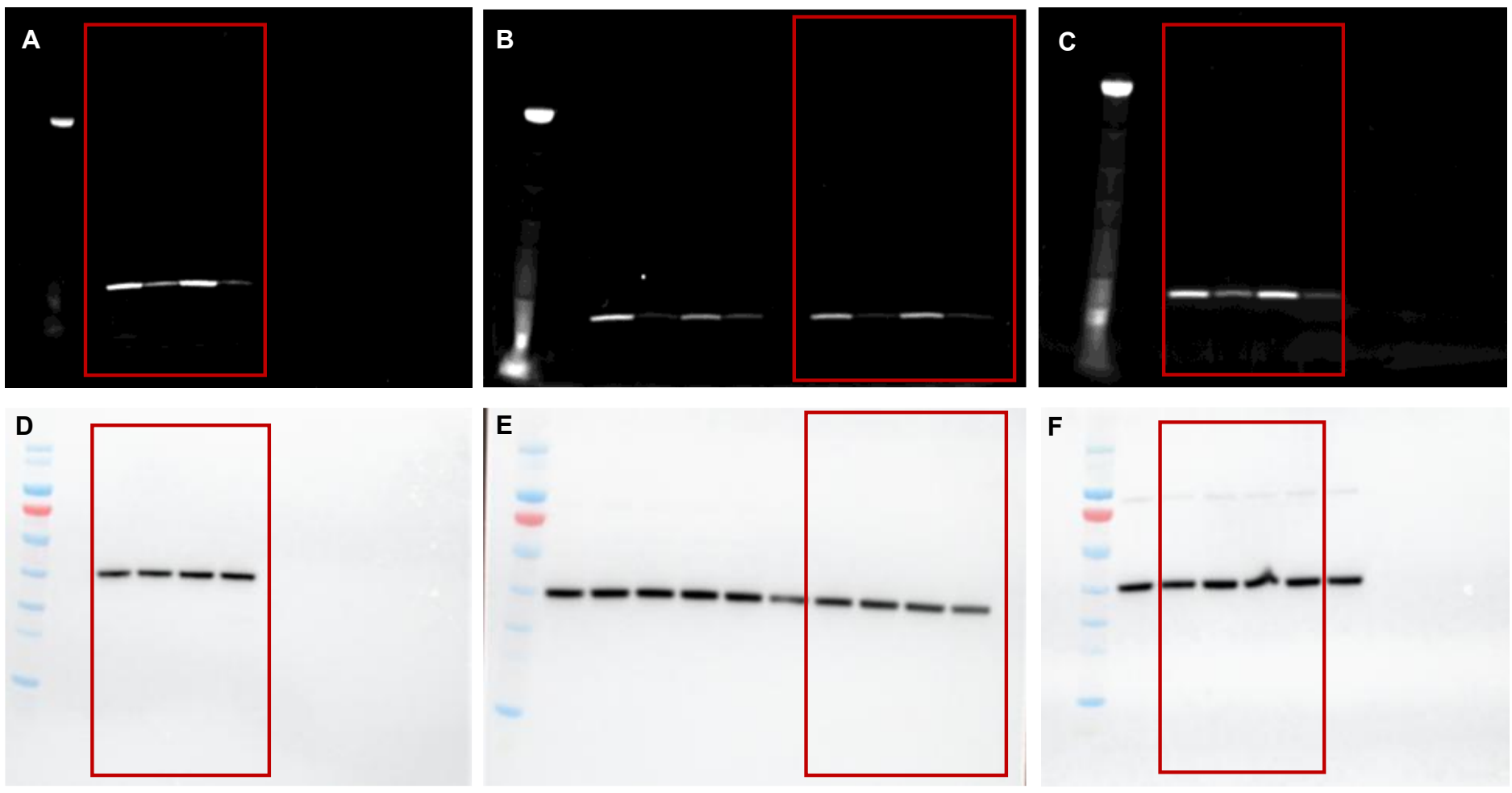

Figure S23. Raw, uncropped images of SDS-PAGE and western blots corresponding to the protein delivery experiments. The sections cropped for presentation in the figures are highlighted. SDS-PAGE gel showing 20 µg of lysate loaded per lane for: A) HeLa, B) U2OS, and C) Jurkat cells. Corresponding western blots probed with rabbit monoclonal anti-GAPDH (1:1000, Abcam, ab181602) as a loading control in: D) HeLa, E) U2OS, and F) Jurkat cells. Band intensities used for quantification in the main figures were normalized to GAPDH.

18. General procedure for cargo uptake studies under 4 °C condition:

Cells were seeded and prepared as described above. Protein solutions were diluted in serum-free medium to the desired final concentration and added to the cells, which were then incubated at 4 °C for 1 h to inhibit energy-dependent endocytic uptake. Following incubation, cells were washed three times with calcium- and magnesium-free PBS, detached (for adherent cells), washed three additional times with PBS, and resuspended in 200 µL PBS for flow cytometry analysis. Additional washes with heparin sulfate (HS), performed to remove membrane-bound material, did not significantly affect the measured fluorescence signal and were therefore not adopted in the final washing protocol. Acquisition and gating were performed as described above, and untreated cells were used as negative controls.

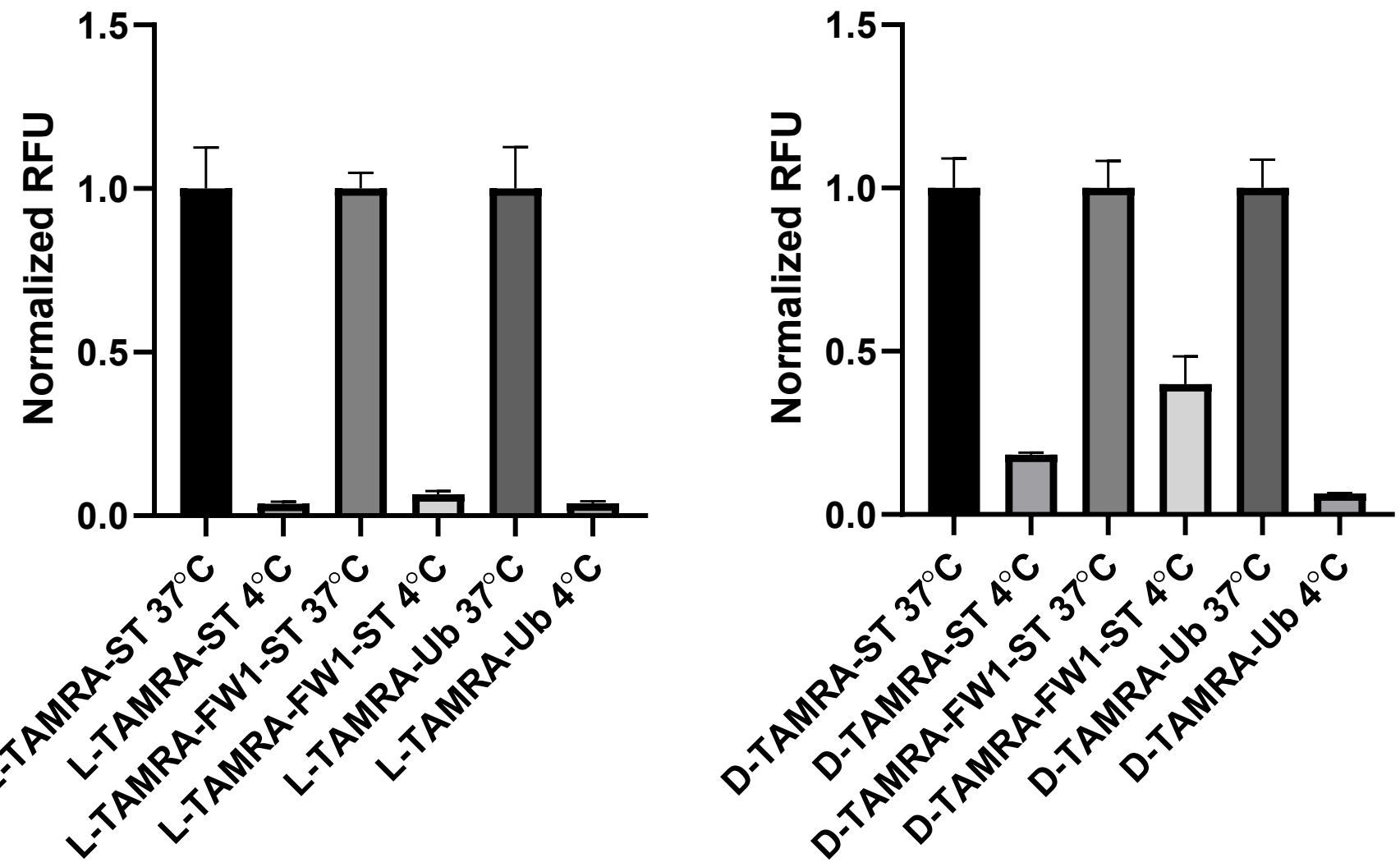

Figure S24. Flow cytometry analysis of TAMRA fluorescence intensities reporting the cellular uptake of each cargo and its mirror-image analogue in U2OS at 2 μM following 1 hour of incubation at 37°C and 4°C.

19. General procedure for cargo uptake studies with endocytosis inhibitor, chlorpromazine:

Cells were seeded and prepared as described above. Prior to protein addition, cells were pre-treated with 20 μM chlorpromazine (CPZ) in serum-free medium for 30 min at 37 °C to inhibit clathrin-mediated endocytosis. Protein solutions were then added at the desired final concentration in the continued presence of CPZ, and cells were incubated for 1 h at 37 °C. Following incubation, cells were washed three times with calcium- and magnesium-free PBS, detached if adherent, washed three additional times with PBS, and resuspended in 200 μL PBS for flow cytometry analysis. Acquisition and gating were performed as described above, with untreated cells and cells treated with protein cargos alone serving as controls.

20. General procedure for cargo uptake studies with macropinocytosis inhibitor, 5-(N-Ethyl-N-isopropyl)amiloride (EIPA):

Cells were seeded and prepared as described above. Prior to protein addition, cells were pre-treated with 25 μM 5-(N-Ethyl-N-isopropyl)amiloride in serum-free medium for 25 min at 37 °C to inhibit macropinocytosis. Protein solutions were then added at the desired final concentration in the continued presence of EIPA, and cells were incubated for 1 h at 37 °C. Following incubation, cells were washed three times with calcium- and magnesium-free PBS, detached if adherent, washed three additional times with PBS, and resuspended in 200 μL PBS

for flow cytometry analysis. Acquisition and gating were performed as described above, with untreated cells and cells treated with protein cargos alone serving as controls.

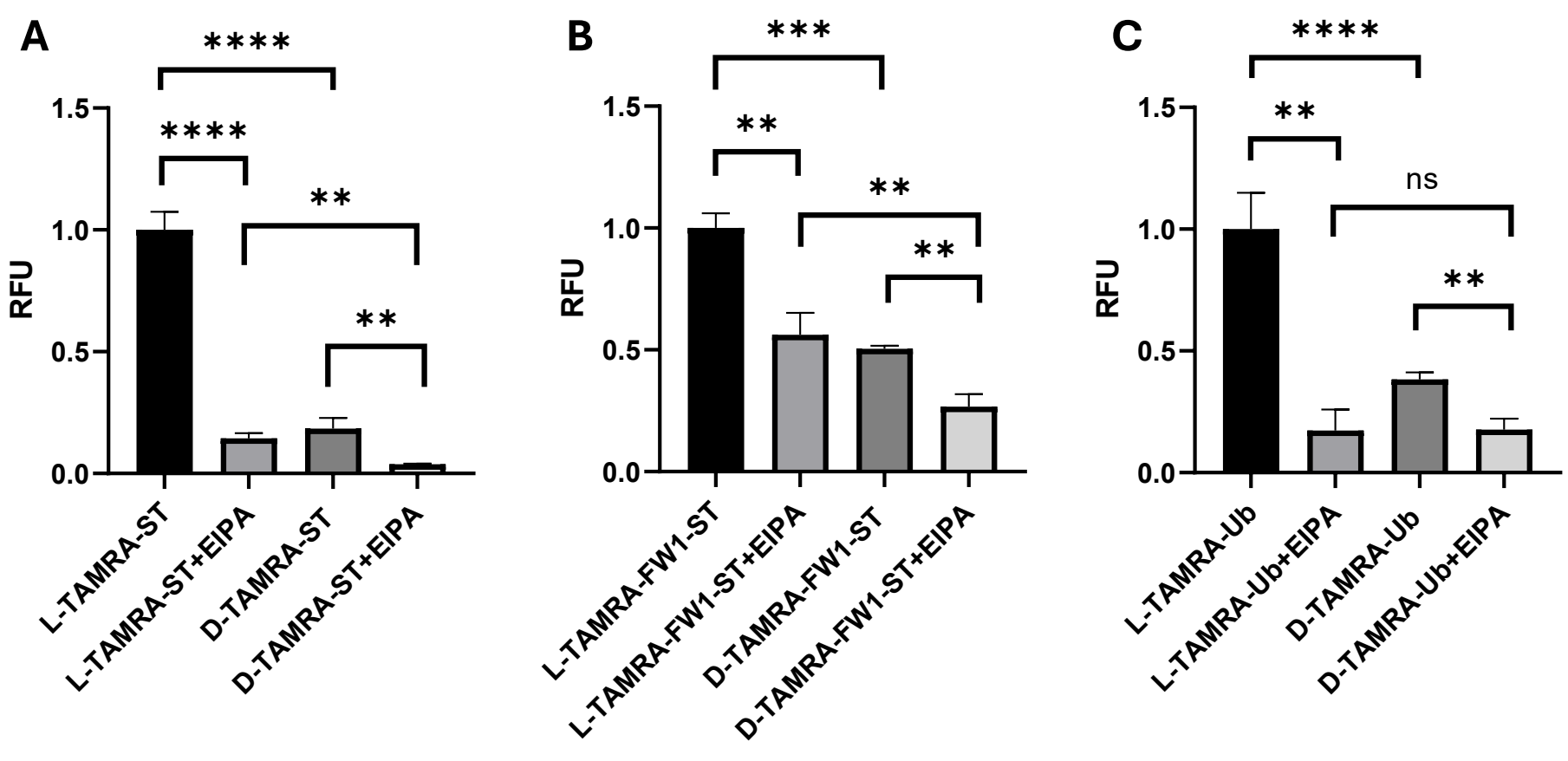

Figure S25. Flow cytometry analysis of TAMRA fluorescence intensities reporting the cellular uptake of each cargo and its mirror-image analogue in U2OS at 2 μM following 1 hour of incubation with micropinocytosis inhibitor, EIPA. A) Quantification of uptake of **L**-and **D-TAMRA-ST**, B) Quantification of uptake of **L**-and **D-TAMRA-FW1-ST**, C) Quantification of uptake of **L**-and **D-TAMRA-Ub**. Data is presented as mean $\pm$SD ($n = 3$). Significance is indicated as: ns = not significant ($p \geq 0.05$), * $p < 0.05$, ** $p < 0.01$, *** $p < 0.001$, **** $p < 0.0001$.

21. Fluorescence characterization of TAMRA-ST and Ub conjugates and GSH Reduction Assay:

To determine whether the DABCYL moiety present on the CPP quenches TAMRA fluorescence and whether this quenching can be reversed under reducing conditions, fluorescence measurements were performed for the L- and D-configured TAMRA-ST-CPP and TAMRA-Ub-CPP constructs. Measurements were carried out in 96-well plates using serial dilutions over a concentration range of 10 μM to 0.156 μM. Prior to fluorescence analysis, sample concentrations were determined using NanoDrop to ensure equal construct concentrations, and TAMRA absorbance was verified to confirm comparable fluorophore loading. Fluorescence emission spectra of TAMRA were recorded from 530 to 640 nm using excitation at 555 nm, and the maximum emission intensity at 581 nm was extracted and plotted as a function of concentration.

For each construct, two parallel sample sets were analyzed: TAMRA-cargo-CPP and TAMRA-cargo-CPP incubated with glutathione (GSH, 5 mM). The GSH-treated samples were incubated at 37 °C for 1 h to promote reduction of the disulfide bond connecting the TAMRA-labeled cargo to the CPP-DABCYL moiety. Fluorescence emission spectra were then recorded under identical conditions, and the fluorescence intensity at 581 nm was used to assess recovery of TAMRA fluorescence upon reduction. For the TAMRA-ST-CPP constructs, only concentrations up to 2.5 µM are shown, as higher concentrations produced saturated fluorescence signals.

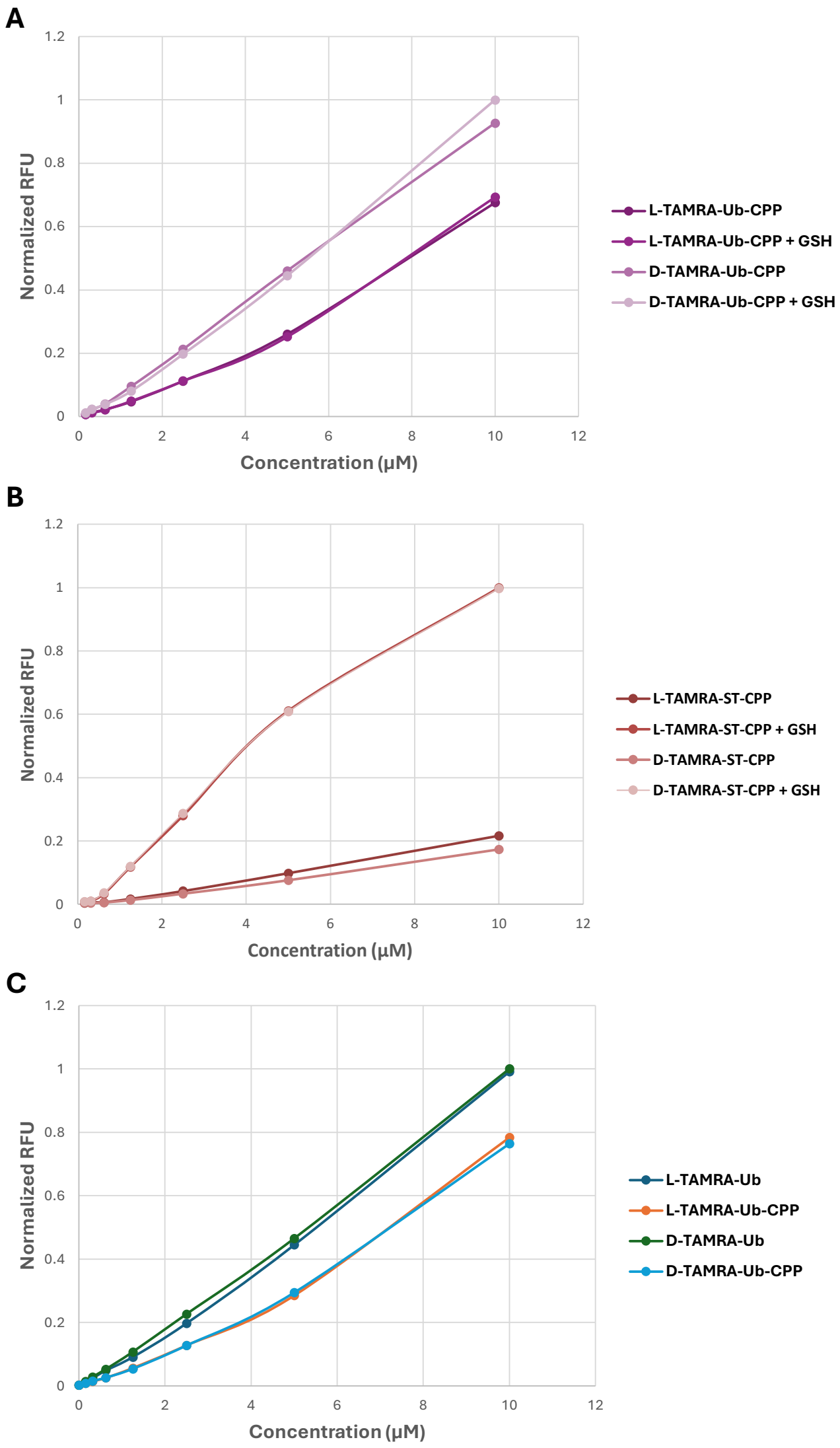

Figure S26. Fluorescence intensity plotted as a function of concentration for TAMRA-labeled ST-CPP and Ub-CPP constructs in the presence or absence of glutathione (GSH). A) **L-TAMRA-Ub**, **L-TAMRA-Ub-CPP**, **D-TAMRA-Ub** and **D-TAMRA-Ub-CPP**, B) **L-TAMRA-ST-CPP** and **D-TAMRA-ST-CPP**, and C) **L-TAMRA-Ub-CPP** and **D-TAMRA-Ub-CPP.**

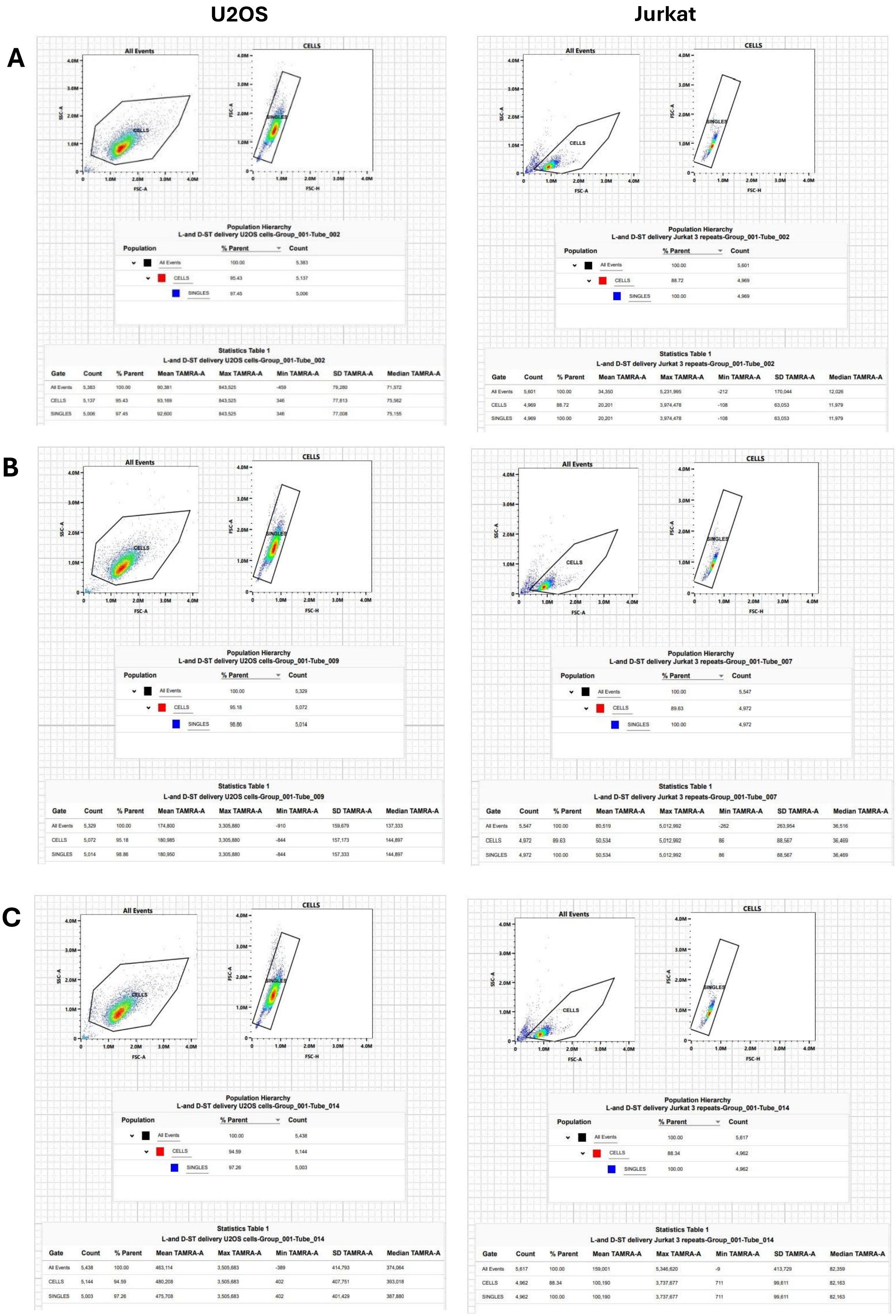

Figure S27. Flow cytometry data of TAMRA fluorescence intensities reporting the cellular uptake of **L-TAMRA-ST-CPP** in U2OS and Jurkat cells at concentrations of: (A) 0.5 µM, (B) 1 µM and (C) 2 µM following 1 hour of incubation.

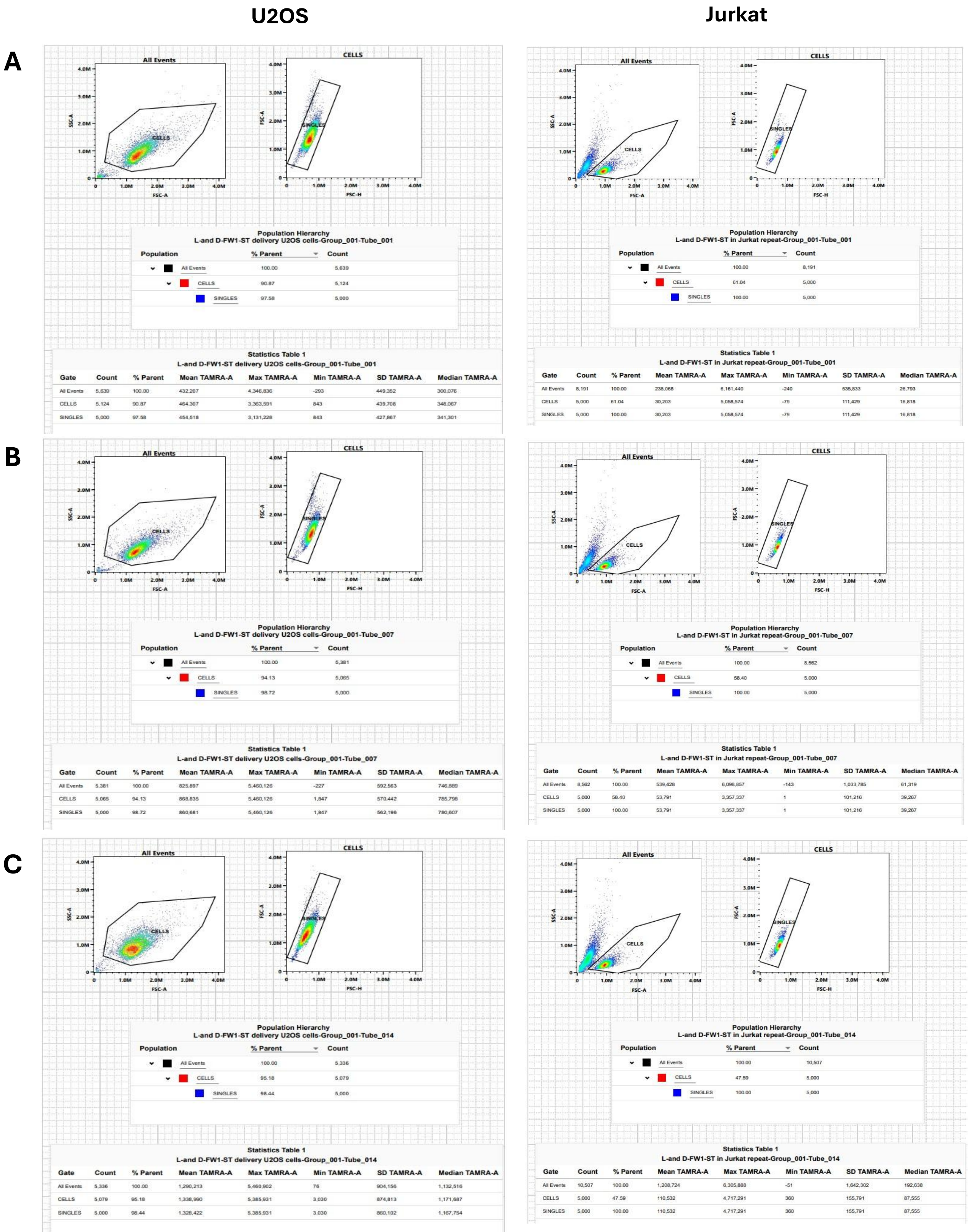

Figure S28. Flow cytometry data of TAMRA fluorescence intensities reporting the cellular uptake of L-TAMRA-FW1-ST-CPP in U2OS and Jurkat cells at concentrations of: (A) 0.5 µM, (B) 1 µM and (C)) 2 µM following 1 hour of incubation.

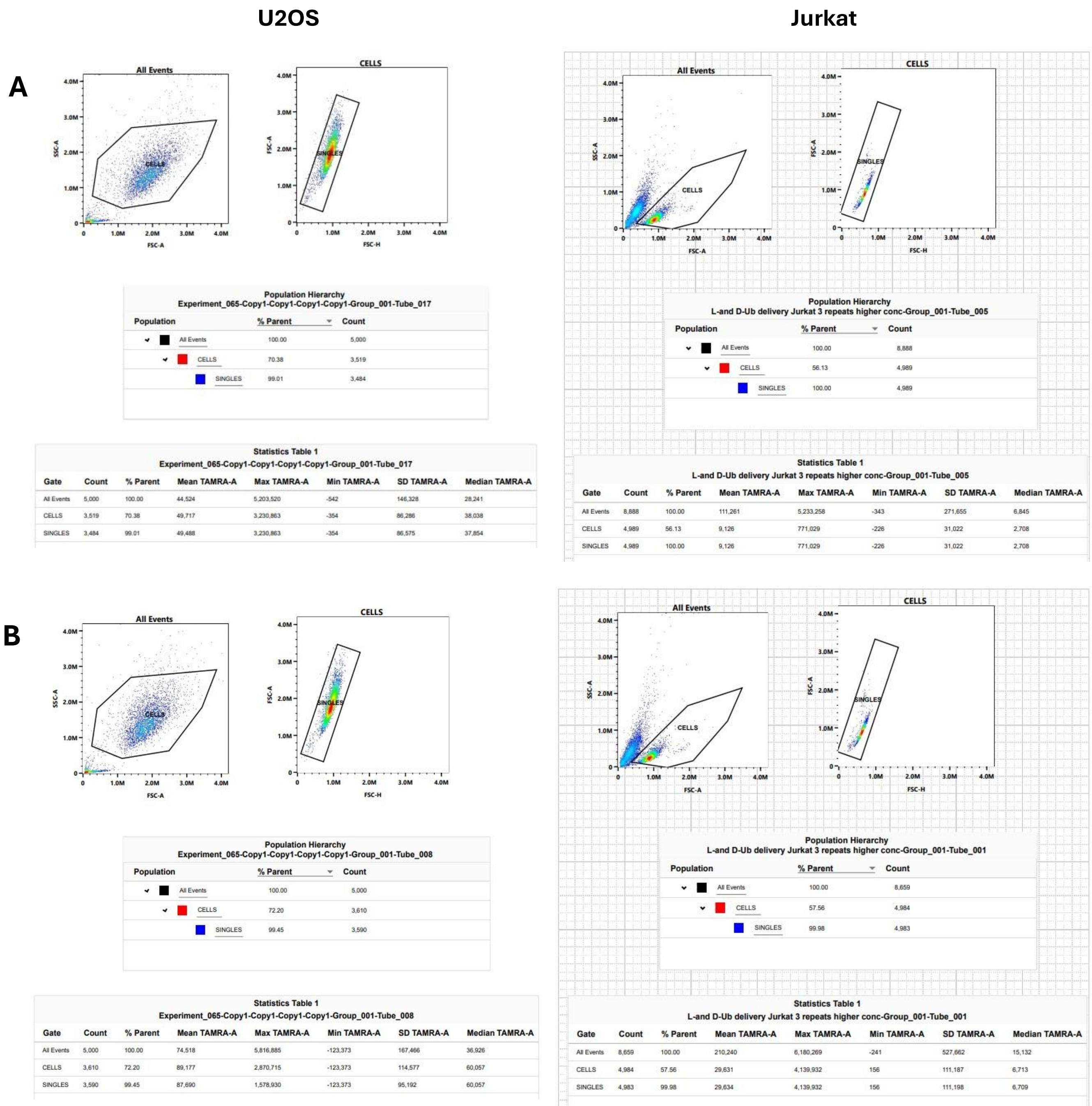

A – U2OS

Population Hierarchy
Experiment_065-Copy1-Copy1-Copy1-Copy1-Group_001-Tube_017

Population	% Parent	Count
All Events	100.00	5,000
CELLS	70.38	3,519
SINGLES	99.01	3,484

Statistics Table 1
Experiment_065-Copy1-Copy1-Copy1-Copy1-Group_001-Tube_017

Gate	Count	% Parent	Mean TAMRA-A	Max TAMRA-A	Min TAMRA-A	SD TAMRA-A	Median TAMRA-A
All Events	5,000	100.00	44,524	5,203,520	-542	146,328	28,241
CELLS	3,519	70.38	49,717	3,230,863	-354	86,286	38,038
SINGLES	3,484	99.01	49,488	3,230,863	-354	86,575	37,854

A – Jurkat

Population Hierarchy
L-and D-Ub delivery Jurkat 3 repeats higher conc-Group_001-Tube_005

Population	% Parent	Count
All Events	100.00	8,888
CELLS	56.13	4,989
SINGLES	100.00	4,989

Statistics Table 1
L-and D-Ub delivery Jurkat 3 repeats higher conc-Group_001-Tube_005

Gate	Count	% Parent	Mean TAMRA-A	Max TAMRA-A	Min TAMRA-A	SD TAMRA-A	Median TAMRA-A
All Events	8,888	100.00	111,261	5,233,258	-343	271,655	6,845
CELLS	4,989	56.13	9,126	771,029	-226	31,022	2,708
SINGLES	4,989	100.00	9,126	771,029	-226	31,022	2,708

B – U2OS

Population Hierarchy
Experiment_065-Copy1-Copy1-Copy1-Copy1-Group_001-Tube_008

Population	% Parent	Count
All Events	100.00	5,000
CELLS	72.20	3,610
SINGLES	99.45	3,590

Statistics Table 1
Experiment_065-Copy1-Copy1-Copy1-Copy1-Group_001-Tube_008

Gate	Count	% Parent	Mean TAMRA-A	Max TAMRA-A	Min TAMRA-A	SD TAMRA-A	Median TAMRA-A
All Events	5,000	100.00	74,518	5,816,885	-123,373	167,466	36,926
CELLS	3,610	72.20	89,177	2,870,715	-123,373	114,577	60,057
SINGLES	3,590	99.45	87,690	1,578,930	-123,373	95,192	60,057

B – Jurkat

Population Hierarchy
L-and D-Ub delivery Jurkat 3 repeats higher conc-Group_001-Tube_001

Population	% Parent	Count
All Events	100.00	8,659
CELLS	57.56	4,984
SINGLES	99.98	4,983

Statistics Table 1
L-and D-Ub delivery Jurkat 3 repeats higher conc-Group_001-Tube_001

Gate	Count	% Parent	Mean TAMRA-A	Max TAMRA-A	Min TAMRA-A	SD TAMRA-A	Median TAMRA-A
All Events	8,659	100.00	210,240	6,180,269	-241	527,662	15,132
CELLS	4,984	57.56	29,631	4,139,932	156	111,187	6,713
SINGLES	4,983	99.98	29,634	4,139,932	156	111,198	6,709

Figure S29. Flow cytometry data of TAMRA fluorescence intensities reporting the cellular uptake of (A) **L-TAMRA-Ub-CPP** and (B) **D-TAMRA-Ub-CPP** in U2OS and Jurkat cells at a concentration of 2 μM following 1 hour of incubation.
